# Thermotropic Liquid-Crystalline and Light-Emitting Properties of Bis(4-aalkoxyphenyl) Viologen Bis(triflimide) Salts

**DOI:** 10.3390/molecules25102435

**Published:** 2020-05-23

**Authors:** Pradip K. Bhowmik, Muhammed Kareem M. Al-Karawi, Shane T. Killarney, Erenz J. Dizon, Anthony Chang, Jongin Kim, Si L. Chen, Ronald Carlo G. Principe, Andy Ho, Haesook Han, Hari D. Mandal, Raymond G. Cortez, Bryan Gutierrez, Klarissa Mendez, Lewis Sharpnack, Deña M. Agra-Kooijman, Michael R. Fisch, Satyendra Kumar

**Affiliations:** 1 Department of Chemistry and Biochemistry, University of Nevada Las Vegas, 4505 S. Maryland Parkway Box 454003, Las Vegas, NV 89154-4003, USA; malkarawi@outlook.com (M.K.M.A.-K.); shanekillarney126@gmail.com (S.T.K.); dizone2@gmail.com (E.J.D.); Changa14@unlv.nevada.edu (A.C.); kimj80@unlv.nevada.edu (J.K.); chens19@unlv.nevada.edu (S.L.C.); princr1@unlv.nevada.edu (R.C.G.P.); hoa3@unlv.nevada.edu (A.H.); hanh3@unlv.nevada.edu (H.H.); 2 Department of Biology and Chemistry, Texas A & M International University, 5201 University Blvd., Laredo, TX 78041, USA; hmandal@tamiu.edu (H.D.M.); Raymond_cortez@dusty.tamiu.edu (R.G.C.); bryangtz11@hotmail.com (B.G.); klarissa11@live.com (K.M.); 3 Department of Earth Science, 1006 Webb Hall, University of California, Santa Barbara, CA 93106, USA; lsharpnack@geol.ucsb.edu; 4 Advanced Materials and Liquid Crystal Institute, Kent State University, Kent, OH 44242, USA; dagrako@kent.edu; 5 College of Aeronautics and Engineering, Kent State University, Kent, OH 44242, USA; mfisch@kent.edu; 6 Division of Research, University at Albany, Albany, NY 12222, USA; satyenkumar@albany.edu

**Keywords:** extended viologens, Zincke salt, metathesis reaction, ionic liquid crystals, thermotropic, smectic phase A, differential scanning calorimetry, polarizing optical microscopy, X-ray diffraction, thermogravimetric analysis

## Abstract

A series of bis(4-alkoxyphenyl) viologen bis(triflimide) salts with alkoxy chains of different lengths were synthesized by the metathesis reaction of respective bis(4-alkoxyphenyl) viologen dichloride salts, which were in turn prepared from the reaction of Zincke salt with the corresponding 4-n-alkoxyanilines, with lithium triflimide in methanol. Their chemical structures were characterized by ^1^H and ^13^C nuclear magnetic resonance spectra and elemental analysis. Their thermotropic liquid-crystalline (LC) properties were examined by differential scanning calorimetry, polarizing optical microscopy, and variable temperature X-ray diffraction. Salts with short length alkoxy chains had crystal-to-liquid transitions. Salts of intermediate length alkoxy chains showed both crystal-to-smectic A (SmA) transitions, T_m_s, and SmA-to-isotropic transitions, T_i_s. Those with longer length of alkoxy chains had relatively low T_m_s at which they formed the SmA phases that persisted up to the decomposition at high temperatures. As expected, all of them had excellent thermal stabilities in the temperature range of 330–370 °C. Their light-emitting properties in methanol were also included.

## 1. Introduction

**Figure 1:**
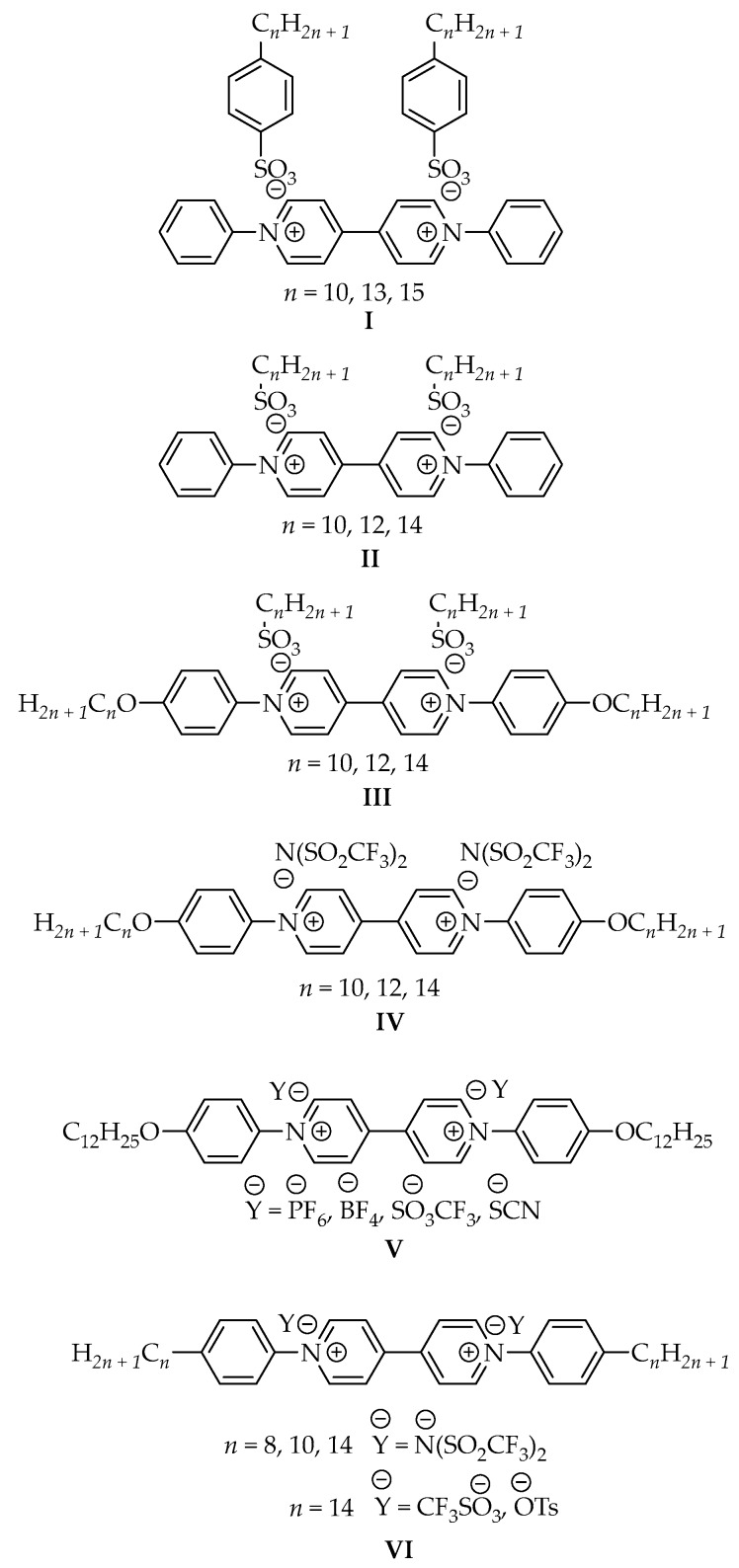
Chemical structures of extended viologen salts that exhibit smectic A (SmA) phases.

Thermotropic ionic liquid crystals (ILCs) are an important class of materials that combine the properties of both ionic liquids and liquid crystals. The liquid-crystalline (LC) phases offer many advantages over the liquid phases. For example, ion conduction is significantly enhanced in smectic A (SmA) and columnar phases as compared with the isotropic liquid phases. The unique properties of ILCs make them suitable for many technological applications including display technology, solar cells, ion conductors in batteries, and templates for the synthesis of nanomaterials [1,2,3,4,5,6,7,8,9,10,11,12,13,14]. They are even considered as organized reaction media in which many organic reactions including Diels–Alder reactions; intramolecular Diels–Alder reactions can be performed with highly regio- and chemoselective reactions, with the additional advantage of being recycled [15,16,17]. They are usually composed of varied suitably modified organic cations and organic and inorganic anions. Among the common cations are quaternary ammonium, quaternary phosphonium, pyrrolidinium, piperidinium, guanidinium, imidazolium, pyridinium pyrazolium, triazolium, among other cations and the common anions are Br^−^, NO_3_^−^, BF_4_^−^, CF_3_SO_3_^−^,PF_6_^−^, ClO_4_^−^, N(SO_2_CF_3_)_2_^−^, OTs^−^, ReO_4_^−^, among other anions. These are examples of monocations and monoanions employed for the synthesis of ILCs [1,2,3,4,5,6,7,8,9,10,11,12,13,14]. Additionally, they can be versatile in their chemical architectures. They can be dicationic, tricationic, and multicationic in their makeups [18,19,20,21,22,23,24,25,26,27,28,29,30]. For example, viologens are examples of dicationic salts that are symmetric (alkyl groups similar) and exhibit LC phases (*n* = 6, 7, 8, 14–20) even at room temperature [31,32,33]. Asymmetric (alkyl groups dissimilar) types with triflimides as counterions exhibit smectic LC phases with a wide range of stability that range from as low as 0 °C to as high as 146 °C. Consequently, they have increased range**s** of stability of LC phases as compared with those for symmetric viologen salts [34]. In the further development of ILCs, there are several reports in the literature on extended viologen salts **(**I–VI**),** in which viologen moiety is elongated with the phenyl residue**,** as shown in Figure 1, and also display smectic LC phases [35,36,37].

**Scheme 1:**
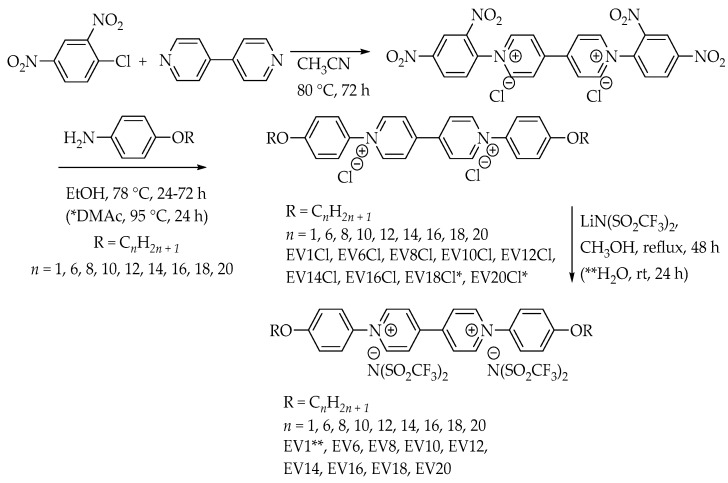
Synthetic routes for the preparation of extended viologen bis(triflimide) salts (EVn). Note that the compounds *n* = 10, 12, and 14 corresponding to structure IV were reported in [36].

As a continuation of our research efforts on ILCs, herein, we describe the synthesis of a series of extended viologen salts with bis(triflimide) (*n* = 1, 6, 8, 10, 12, 14, 16, 18, and 20), where n denotes the carbon atoms in the alkoxy chain. We determine their chemical structures by ^1^H and ^13^C NMR spectra, as well as elemental analysis, and the characterization of their thermotropic LC properties by several experimental techniques including differential scanning calorimetry (DSC), polarizing optical microscopy (POM), and variable temperature X-ray diffraction (VT-XRD) studies. Their thermal stabilities by thermogravimetric analysis (TGA) are also included. The general structures and designations for these synthesized extended viologen salts, and their synthetic routes are shown in Scheme 1. The LC properties of these salts with these triflimide anions enable one to establish the structure–property relationship of this important class of ILCs. Additionally, in contrast to other anions, the reports of ILCs on triflimide anions are relatively less studied [24,31,32,33,34,38,39,40] which give the impetus for this study.

## 2. Results and Discussion

In this study, a series of extended viologen bis(triflimide) salts (EVn) with variable alkoxy chains were synthesized, characterized for their chemical structures, and further characterized for their thermotropic LC properties using DSC, POM, and VT-XRD techniques. The thermal stabilities of the salts were determined using TGA technique. Their light-emitting properties including their chloride precursors in methanol were studied using UV-Vis and photoluminescence spectrometer.

### 2.1. Synthesis of Bis(4-n-alkoxyphenyl)-4,4′-bipyridinium Bis(triflimide) Salts (EV1, EV6, EV8, EV10, EV12, EV14, EV16, EV18, and EV20)

The 4-n-alkoxyanilines were prepared in accordance with the modified literature procedure [41]. The bis(4-alkoxyphenyl)-4,4′-bipyridinium dichloride (EV1Cl, EV6Cl, EV8Cl, EV10Cl, EV12Cl, EV14Cl, EV16Cl, EV18Cl, and EV20Cl) with alkyl chains of different lengths were synthesized as shown in Scheme 1 [42,43,44]. The synthetic route was based on a two-step procedure involving the aromatic nucleophilic substitution between the 1-chloro-2,4-dinitrobenzene and 4,4′-bipyridine to give the Zincke salt and its subsequent ANRORC (anionic ring opening and ring closing) reaction with the corresponding 4-n-alkoxyanilines. For the preparation of Zincke salt, the usual solvent acetonitrile was used. For the ANRORC reaction, the usual solvent ethanol was used except for EV18Cl and EV20Cl. In these cases, DMAc was used for the better solubility of these long carbon chains 4-n-alkoxyanilines [36]. The structures of chlorides salts of shorter carbon chains, up to *n* = 12, were established from their ^1^H and ^13^C NMR spectra obtained in CD_3_OD (Figures S1–S5). For the longer carbon chains, *n* = 14–20, we were able to verify their chemical structures from ^1^H NMR spectra in CD_3_OD only (Figures S6–S9). Then. these chlorides salts were converted to bis(triflimides) salts by the metathesis reaction with lithium triflimides in a common solvent methanol [31,32,33]. For EV1, the metathesis reaction was carried out in water. Then, the bis(triflimide) salts with shorter carbon chains, up to *n* = 12, were characterized analyzing the ^1^H and ^13^C-NMR spectra obtained in CD_3_OD (Figures S10–S14), while those with longer carbon chains, *n* = 14, 16, 18, 20, were characterized analyzing the ^1^H and ^13^C NMR spectra obtained in *d_6_-*DMSO (Figures S15–S18). Their purity was also determined from their elemental analysis.

### 2.2. Thermotropic LC Properties of EVn by DSC, POM, and VT-XRD 

**Figure 2:**
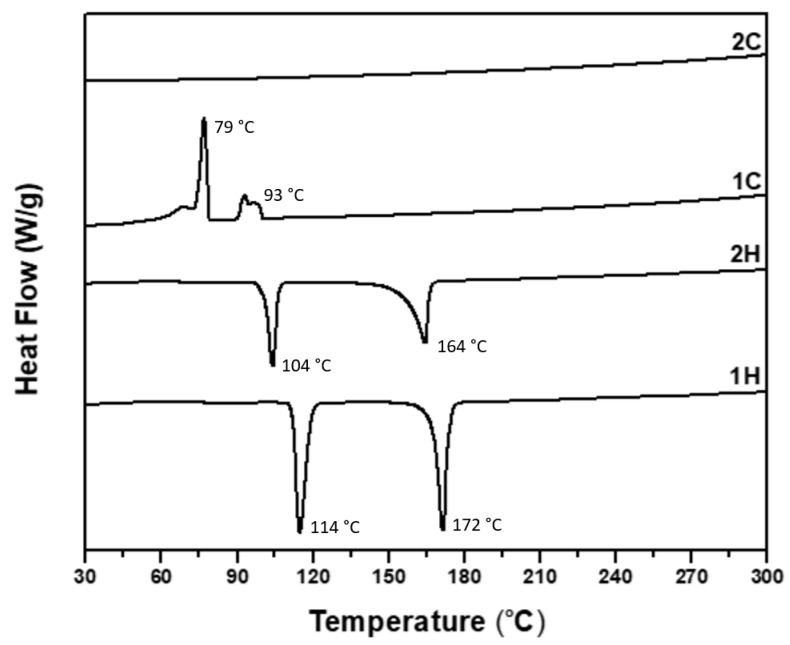
Differential scanning calorimetry (DSC) thermograms of EV1 obtained at heating and cooling rates of 10 °C /min.

Figure 2 displays the DSC thermograms of EV1 in the heating and cooling cycles. It clearly shows two endotherms in each of the two heating cycles. The peak maxima of the endotherms, as well as heat of enthalpies in the second heating cycle, were slightly lower than those in the first heating cycle [45,46,47,48,49].

**Figure 3:**
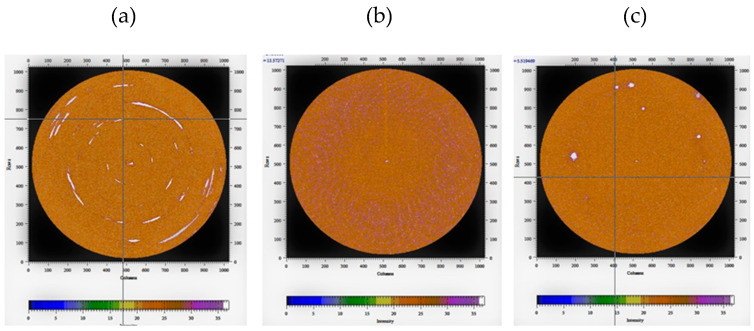
The X-ray diffraction patterns of EV1. (**a**) Crystalline phase at room temperature; (**b**) Isotropic phase taken at 200 °C; (**c**) Incipient crystalline phase at 160 °C; on cooling from isotropic phase. It can be seen that this compound scatters X-ray poorly.

In the first cooling cycle, there were cooling exotherms with large supercooling and decreased heat of enthalpies. In the second cooling cycle, there were no exotherms which suggested that no crystallization occurred under the experimental conditions used. Figure 3 shows diffraction patterns of EV1 that indicated that it has crystalline phase at room temperature, an isotropic phase of diffuse scattering rings at 200 °C, and incipient crystalline phase at 160 °C on slow cooling from the isotropic phase. These results suggested that the high-temperature endotherm corresponded to crystal-to-liquid transition, T_m_, at 164 °C. The low-temperature endotherm corresponded to crystal-to-crystal transition. The POM studies also corroborated these statements. Thus, EV1 did not form any LC phase.

Figure S19 shows the DSC thermograms of EV6 in its heating and cooling cycles. In the first heating cycle, it showed three endotherms located at 81, 93, and 114 °C. In the second heating cycle, the absence of low-temperature endotherms and only the presence of a large endotherm at 105 °C with the increased heat of enthalpy suggested that low-temperature endotherms were related to crystal-to-crystal transition and high-temperature was related to crystal-to-liquid transition. The presence of cooling exotherm in each of the cooling cycles suggested that the crystallization from the isotropic liquid occurred under the experimental conditions used. The first cooling and second cooling exotherms underwent a relatively low degree of supercooling of 14 and 6 °C, respectively.

The POM studies also corroborated by the fact that it formed the typical crystal texture. Thus, similar to EV1, EV6 did not form any LC phase.

**Figure 4:**
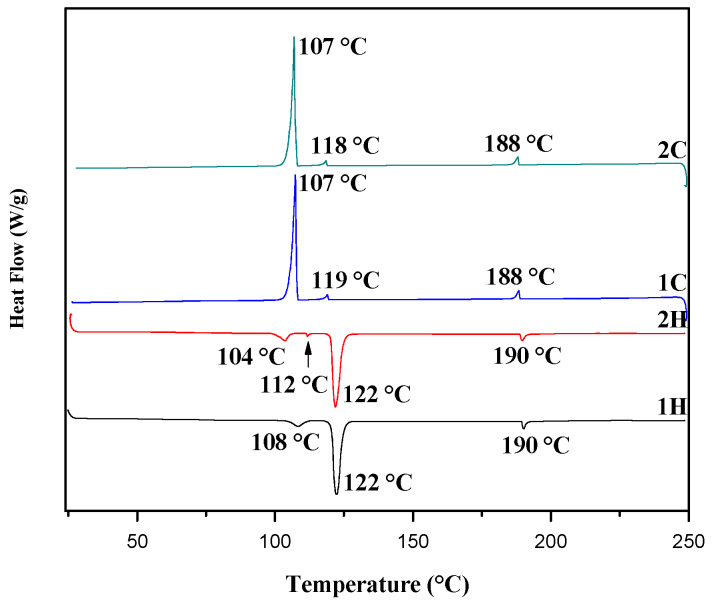
DSC thermograms of EV8 obtained at heating and cooling rates of 10 °C /min.

**Figure 5:**
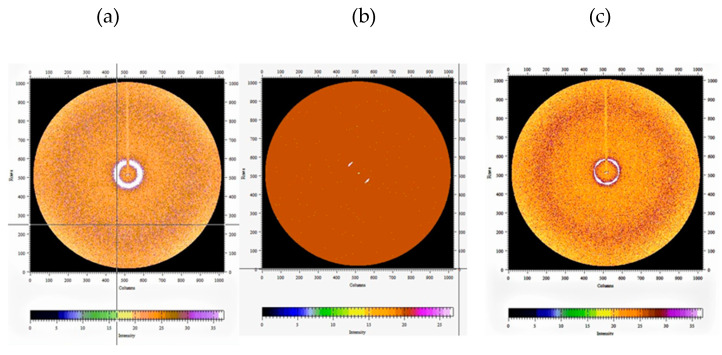
The X-ray diffraction patterns of EV8. (**a**) Isotropic phase taken at 200 °C; (**b**) Sharp inner arcs of SmA taken at 190 °C; (**c**) SmA of inner ring sharp and outer ring diffuse at 180 °C.

**Figure 6:**
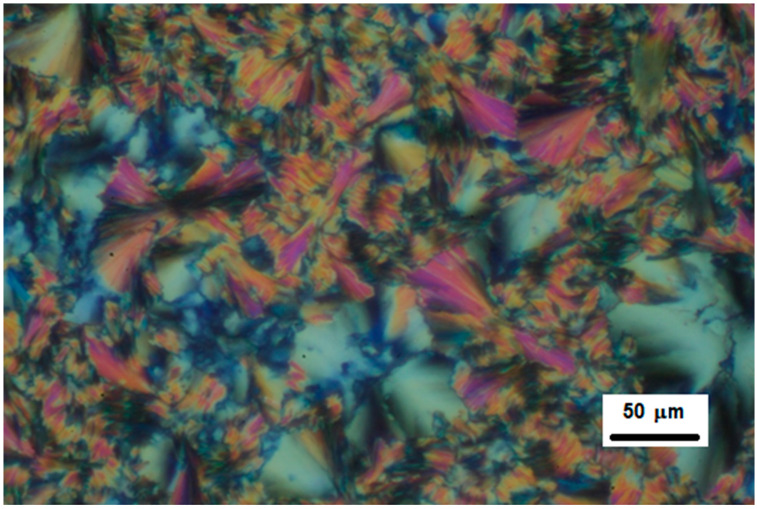
The focal-conic texture of EV8 obtained at 160 °C on cooling from the isotropic phase (magnification 200×).

Figure 4 shows the DSC thermograms of EV8 in its heating and cooling cycles. In the first heating cycle it showed three endotherms located at 108, 122, and 190 °C. In the second heating cycle, there were four endotherms located at 104, 112, 122, and 190 °C. In each of the cooling cycles, there were three exotherms. In conjunction with VT-XRD studies, it was found that the endotherm at 190 °C corresponded to SmA-to-isotropic transition, T_i_, and that at 122 °C it corresponded to crystal-to-SmA transition, T_m_, since it showed diffraction patterns of diffuse rings at 200 °C, inner arcs of SmA at 190 °C, and sharp inner ring and outer diffuse ring at 180 °C (Figure 5). The endotherm(s) prior to the T_m_ corresponded to crystal-to-crystal transition(s). The exotherm prior to the recrystallization exotherm in each of the cooling cycles presumably corresponded to the transition from the SmA to another (monotropic) mesophase. Interestingly, both T_m_ and T_i_ underwent very little supercooling in the cooling cycles which was in stark contrast to many ILCs [50,51,52,53,54]. Additionally, the POM studies also indicated that EV8 formed an SmA phase, since its optical texture exhibited the so-called focal-conic texture taken at 160 °C on cooling from the isotropic phase (Figure 6) [45,46,47,48,49]. Thus, these results suggested that EV8**,** unlike EV1 and EV6, showed an SmA phase after T_m_ and a T_i_ leading to the LC phase range of 68 °C. The analogous salt containing n-octyl group has a T_m_ at 103 °C and Ti at 180 °C resulting in the LC phase range of 77 °C [37].

**Figure 7:**
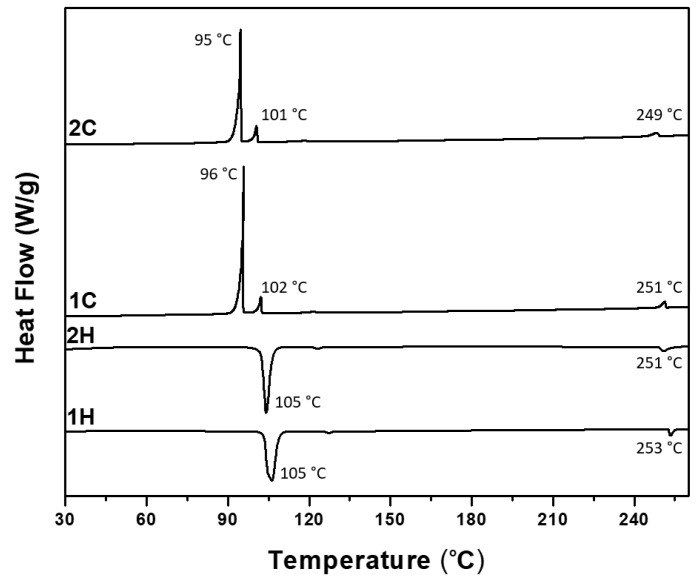
DSC thermograms of EV10 obtained at heating and cooling rates of 10 °C /min.

Figure 7 shows the DSC thermograms of EV10 in its heating and cooling cycles. In the first heating cycle, it showed three endotherms located at 106, 127, and 253 °C. In the second heating cycle, there were also three endotherms located at 104, 123, and 251 °C. In each of the cooling cycles, there were four exotherms. In the POM studies, we determined that its T_m_ at 104 °C and T_i_ at 251 °C resulted in an LC phase range of 147 °C and confirmed the results, as reported in the literature [36]. The exotherm, prior to the recrystallization exotherm in each of the cooling cycles, presumably corresponded to the transition from the SmA to another (monotropic) mesophase. The corresponding salt having n-decyl group has a T_m_ at 75 °C and T_i_ at 175 °C resulting in the LC phase range of 100 °C [37]. However, we were at variance with the fact that there was an additional endotherm in between T_m_ and T_i_ in each of the heating cycles, although the nature of this endotherm remains unknown. This endotherm was probably related to the LC-to-LC transition. Similar to EV8, both T_m_ and T_i_ exhibited little supercooling in contrast to many ILCs that undergo large supercooling, also known as hysteresis [50,51,52,53,54,55,56,57].

**Figure 8:**
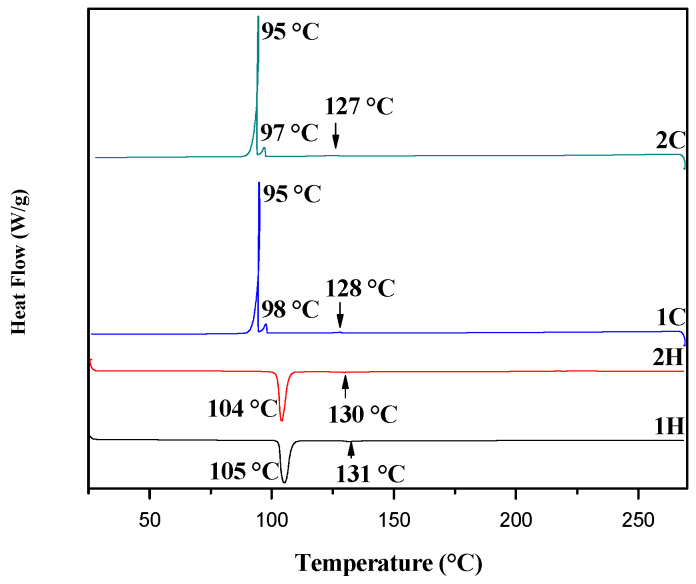
DSC thermograms of EV12 obtained at heating and cooling rates of 10 °C /min.

Figure 8 shows the DSC thermograms of EV12 in its heating and cooling cycles. In the first heating cycle, it showed a large endotherm located at 105 °C and a small endotherm at 131 °C. In the second heating cycle, there was also a large endotherm located at 104 °C and a small endotherm at 130 °C. In each of the cooling cycles, there were three exotherms. In the POM studies, we determined that its large endotherm corresponded to T_m_ at 104 °C which was in excellent agreement as reported in the literature [36]. The exotherm prior to the recrystallization exotherm in each of the cooling cycles presumably corresponded to the transition from the SmA to another (monotropic) mesophase. We tried to confirm its reported T_i_ at 305 °C and found that this transition appeared in the first heating cycle followed by decomposition (Figure S20), since the T_m_ endotherm in the first heating cycle was not reproducible in the second heating cycle. However, we were at variance with the fact that there was an additional endotherm after T_m_ in each of the heating cycles, although the nature of this endotherm remains unknown. Presumably, they were related to the LC-to-LC transition. Unlike EV8 and EV10, EV12 showed T_m_ after which it formed an SmA phase that persisted up to the decomposition temperature at high temperature (vide infra) [45,46,47,48,49].

**Figure 9:**
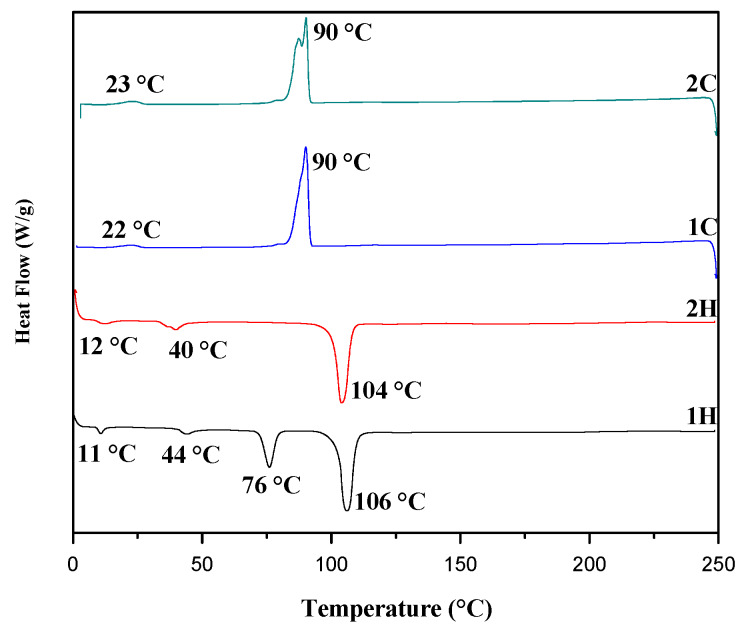
DSC thermograms of EV14 obtained at heating and cooling rates of 10 °C /min.

**Figure 10:**
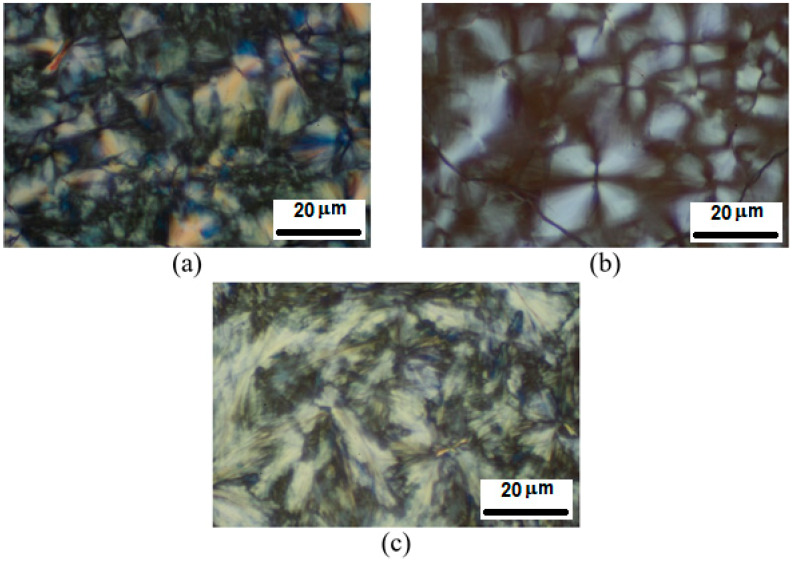
Optical textures of EV16, EV18, and EV20 taken at (**a**) 240, (**b**) 150, and (**c**) 290 °C, respectively, displaying SmA phases (magnification 400×).

Figure 9 shows the DSC thermograms of EV14 in its heating and cooling cycles. In the first heating cycle, it showed four endotherms located at 11, 44, 76, and 106 °C. In the second heating cycle, there were three endotherms located at 12, 44, and 104 °C. The POM studies suggested that the highest-temperature endotherm at 104 °C is its T_m_ after which it transformed into SmA which persisted up to the decomposition at high temperature. This T_m_ was in excellent agreement with that in the literature [36]. However, it did not show T_i_. In contrast, the corresponding salt with n-tetradecyl group has a T_m_ at 80 °C and T_i_ at 320 °C resulting in the LC phase range of 240 °C [37]. Again, we were at variance with the T_i_ at 328 °C which was also determined by POM and not detected by DSC [36]. Additionally, the additional endotherms prior to the T_m_ corresponded to crystal-to-crystal transition which is known as polymorphism [58,59,60,61,62]. It was verified by observations of a highly birefringence texture. There were sharp textural defects and absence of homogeneity in the domains suggesting its crystal phases. The features for the DSC thermograms of EV16, EV18, and EV20 in their heating and cooling cycles were essentially identical (Figures S21–S23) with a minor exception. They showed essentially similar T_m_s (101, 106, and 107 °C, respectively,) similar to EV14 (104 °C), after which they transformed into SmA phases (Figure 10) which persisted up to their decomposition at high temperatures. They also showed the polymorphism phenomenon since there were additional endotherms prior to the T_m_s. In the cases of EV16 and EV20**,** each of them exhibited an exotherm at ca. 260 °C in the first heating cycle in the SmA phase. The reason for this exotherm remains to be explored, although there exists a precedence in the literature [22], such as exotherms in the smectic phases of other ILCs without any definitive answers. However, we speculate the reason for the exotherm was related to conversion of metastable state of smectic phase to stable state of smectic phase, since it appeared only in the first heating cycle.

The thermodynamic properties of phase transition temperatures of EVn determined from DSC measurements and decomposition temperatures from TGA measurements are compiled in Table 1. It shows that EV1 had the highest melting point of 164 °C in the series but no LC property. EV6 had the lower melting point of 105 °C as compared with EV1 and had no LC property, similar to EV1**.** Interestingly, all other members in the series formed SmA phases at their respective T_m_s. EV8 had the highest T_m_ and the rest of the members had essentially similar low T_m_s. EV8 and EV10 also exhibited T_i_s, thus resulting in the LC phase ranges of 68 and 147 °C, respectively. EV12–EV20 formed SmA phases at low T_m_s that persisted up to decomposition temperature at high temperatures. On the one hand, these results can be rationalized by the fact that the short alkyl (alkoxy) chain does not permit the nanosegregation between the ionic groups and hydrophobic groups, and thus prevents the formation layer structures. On the other hand, the long alkyl (alkoxy) chain permits the nanosegregation between the ionic moieties and hydrophobic moieties leading to layered smectic phases, in which ionic layers are alternated with hydrophobic alkoxy/alkyl layers, which is the essential criterion for the formation of ILCs [63,64,65]. The results also suggested that the length of the alkoxy had little effect on T_m_s but a significant effect on the T_i_s, which was in stark contrast to molecular LCs [66]. The longer the alkoxy chain, the higher the temperature at which an LC phase transformed into an isotropic phase and the larger the LC temperature range [67]. These results are in excellent agreement with those of other ILCs including the C_n_mim family (1-alkyl-3-methylimidazolium salts) [54,68,69]. Although triflimide counterion is a large size ion with diffuse electron cloud, it can provide low electrostatic attraction interactions with many cations, thus, leading to many ionic liquids, but it is also capable of forming ILCs with suitably designed cations such as extended viologen cation moieties which are similar to the viologen moieties reported earlier in the literature [31,32,33,34]. Specifically, the thermotropic liquid-crystallinity of the short alkoxy chain, as low as octyloxy in the extended viologen moiety, with this triflimide ion is quite an interesting result in the field of ILCs.

### 2.3. Thermal Stabilities of Extended Viologen Bis(triflimide) Salts (EVn)

**Figure 11:**
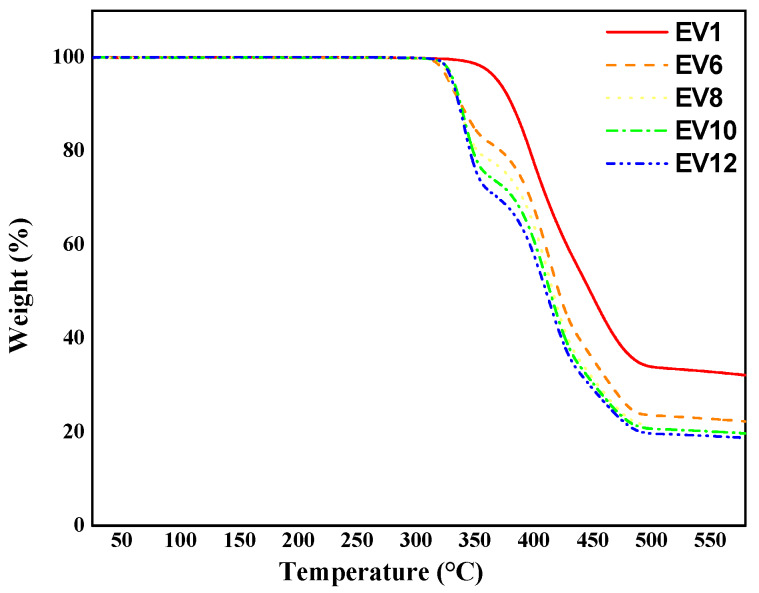
TGA thermograms of EV1–EV12 obtained a heating rate of 10 °C/min in nitrogen.

The stabilities for all extended viologen salts were studied by TGA analyses. They are defined as the temperature (°C) at which a 5% weight loss for each of the salts occurred at a heating rate of 10 °C/min in nitrogen. Despite the presence of flexible alkyl chains, the representative TGA thermograms of five salts were plotted, as shown in Figure 11, and display relatively high thermal stabilities that are in the temperature range of 330–370 °C (Table 1, Figure S24). There were no apparent specific trends along the series. However, EV1 had the highest thermal stability and EV20 had the lowest thermal stability. Triflimide is one of best counterion that imparts the high thermal stability of any ionic liquids, ILCs, and ionic polymers reported in the literature [70,71,72], because it has non-nucleophilic character being the conjugate base of a super acid. Therefore, it causes decomposition of the associated cationic moieties nucleophilically at relatively high temperatures.

### 2.4. Light Emission Properties of EVCl6 and EV6

**Figure 12:**
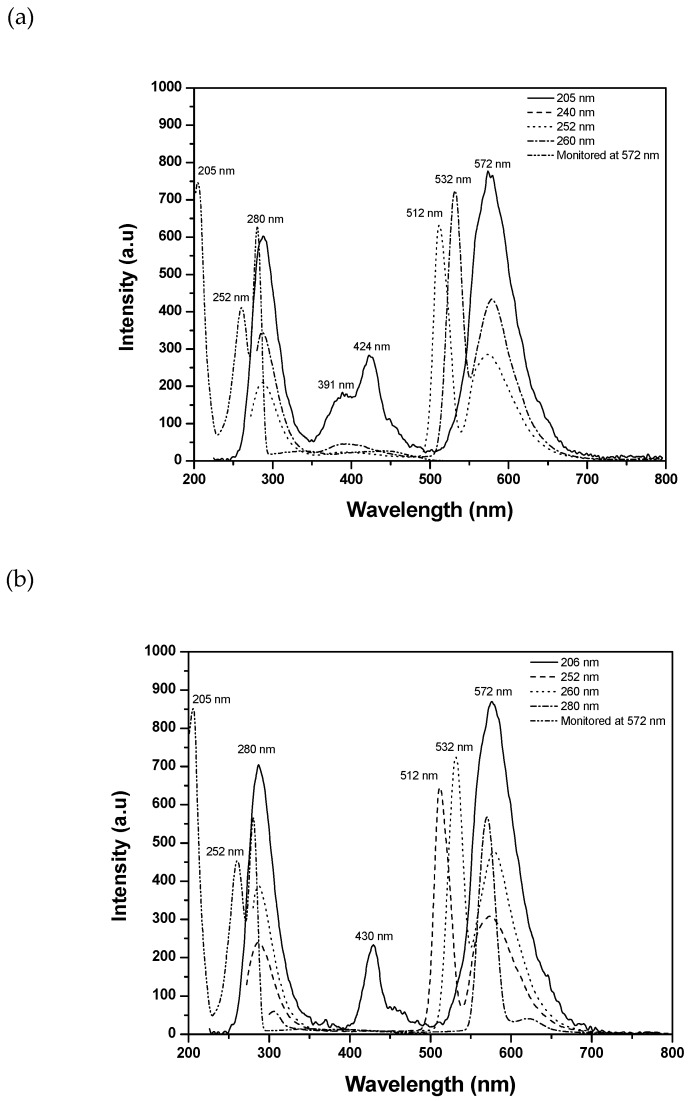
Emission spectra of (**a**) EV6Cl and (**b**) EV6 in methanol at various excitation wavelengths.

All the extended viologens with dichloride and bis(triflimide) showed essentially identical, absorption, excitation, and emission spectra in methanol as measured by UV-Vis and luminescence spectrometer. Figure 12 shows the emission spectra of EV6Cl and EV6 in methanol that are representative of the light emission properties of this class of salts (Figures S25–S27). Each of them showed a major peak at 572 nm when excited at various excitation wavelengths of 205–260 nm. This major emission peak was independent of the counterions (chloride and triflimide) and the length of alkoxy groups attached to the extended viologen moiety.

## 3. Materials and Methods

### 3.1. Instrumentation

The ^1^H and ^13^C nuclear magnetic resonance (NMR) spectra of all the extended viologen bis(triflimide) salts in CD_3_OD or *d_6_-*DMSO were recorded by using VNMR 400 spectrometer operating (Varian Inc., Palo Alto, CA, USA.) at 400 and 100 MHz, at room temperature. Elemental analysis was performed by Atlanta Microlab Inc., (Norcross, GA, USA). Differential scanning calorimetry (DSC) measurements of all the salts were conducted on a TA module DSC Q200 series (TA Instruments, New Castle, DE, USA) in nitrogen at heating and cooling rates of 10 °C/min. The temperature axis of the DSC thermograms was calibrated before use with reference standards of high purity indium and tin. Their thermogravimetric analyses (TGA) were performed using a TGA Q50 instrument (TA Instruments, New Castle, DE, USA) at a heating rate of 10 °C/min, in nitrogen. Optical studies were performed on these viologen salts sandwiched between a standard microscope glass slide and coverslip. The samples were heated and cooled on a Mettler hot-stage (FP82HT) and (FP90) controller (Mettler Toledo, Columbus, OH, USA) and observations of the phases made between crossed polarizers of an Olympus BX51 microscope (Olympus America Inc., New York, NY, USA). In short, salts were heated above their clearing transitions, whenever possible, and cooled at 5 °C/min to room temperature, with brief pauses to collect images and observe specific transitions. In some cases, the salts were heated well above the T_m_s to make thin films, and photomicrographs were taken at specific temperatures. X-ray diffraction studies of salts contained in flame sealed 1 mm quartz capillaries were performed using a Rigaku Screen Machine (Rigacu, Inc., The Woodlands, TX, USA). The salt under study was placed inside the Linkam HFS350X-Cap capillary (Linkam Scientific Instruments, Tadworth, UK) hot-stage 78 mm away from the two-dimensional (2D) detector, with temperature controlled to the accuracy of ± 0.1 °C. A magnetic field of ~2.5 kG was applied to the samples using a pair of samarium cobalt permanent magnets with B nearly parallel to the beam stop. Scattering patterns were collected using a Mercury 3 CCD detector (Rigacu, Inc., The Woodlands, TX, USA) with resolution 1024 × 1024 pixels (size, 73.2 µm × 73.2 µm) and copper K_α_ radiation generated by a microfocus sealed X-ray tube with copper anode (λ = 1.542 Å). The UV-Vis absorption spectra in methanol were recorded with a Varian Cary 3 Bio UV-Vis spectrophotometer (Agilent Technologies, Santa Clara, CA, USA) at rt. Photoluminescence spectra in methanol solutions were recorded with a Perkin–Elmer LS 55 luminescence spectrometer (Perkin Elmer, Akron, OH, USA) with a xenon lamp light source.

### 3.2. Materials

4-Hydoxyacetanilide, *n*-hexyl bromide, *n* -octyl bromide, *n* -decyl bromide, *n* -dodecyl bromide, *n* -tetradecyl bromide, *n* -hexadecyl bromide, *n* -octadecyl bromide, eicosyl bromide, 4,4′-bipyridine, 1-chloro-2,4-dintrobenzene, 4-methoxyaniline, potassium carbonate, sodium hydroxide, and lithium triflimide were purchased from TCI America (Portland, OR, USA) and used as received. For synthesis and purification purposes, reagent grade solvents including acetonitrile, ethanol, methanol, ethyl acetate, *N*,*N*-dimethylacetamide (DMAc), spectral grade methanol were used as obtained from Sigma-Aldrich (Milwaukee, WI, USA).

### 3.3. Synthesis of 4-n-Alkoxyanilines (n = 6, 8, 10, 12, 14, 16, 18, 20)

All the 4-n-alkoxyanilines were prepared according to the slightly modified literature procedure [41]. The modification was the use of acetone instead of *N*,*N*-dimethylformamide in the alkylation of 4-hydroxyacetanilide with the respective n-alkyl bromide followed by hydrolysis of 4-n-alkoxyacetanilide. The overall yields for the synthesis of 4-n-alkoxyamines in two-step reaction were 74%, 60%, 67%, 62%, 845, 805, 85%, and 86%.

### 3.4. Synthesis of Zincke Salt

Zincke salt was prepared from the reaction of 1-chloro-2,4-dinitrobenzene (2.5 equiv) with 4,4′-bipyridine (1 equiv) on heating in acetonitrile according to the procedure described in the literature [42,43].

### 3.5. General Procedure for the Synthesis of Bis(4-alkoxyphenyl)-4,4′-Bipyridinium Dichloride (EV1Cl, EV6Cl, EV8Cl, EV10Cl, EV12Cl, EV14Cl, EV16Cl, EV18Cl, and EV20Cl)

The procedure that was adopted for the synthesis of EV1Cl from the reaction of Zincke salt with 4-methoxyaniline [44], as an example, is as follows: Ethanol was added to 1,1′-bis(2,4-dinitrophenyl)-(4,4′-bipyridine)-1,1′-diium chloride (2.00 g, 3.56 mmol) and 4-methoxyaniline (1.10 g, 8.90 mmol) to form a yellow solution. The reaction mixture was heated on stirring to reflux under nitrogen, for 24 h. At the end of the reaction, it was brought to room temperature. Ethanol was removed from the reaction mixture using a rotary evaporator to yield the reaction products that were, then, dried overnight in vacuum to remove any residual solvent. Then, the desired crude product was obtained by removing 2,4-dintroaniline with plenty of hot acetone and collected by vacuum filtration. Finally, it was purified from recrystallization from methanol yielding (1.44 g, 3.26 mmol) a yellow to dark yellow powder. Similarly, EV6Cl, EV8Cl, EV10Cl, EV12Cl, EV14Cl, and EV16Cl were prepared from the corresponding 4-n-alkoxyanilines and recrystallized from an appropriate solvent or solvent mixtures (vide infra). In the cases of EV18Cl and EV20Cl, the solvent DMAc was used because of better solubility of these amines instead of ethanol according to the procedure reported in the literature [36].

Data for EV1Cl: Yield 92%. δ_H_ (CD_3_OD, 400 MHz, ppm): 9.50 (4H, d, *J* = 7.2 Hz), 8.90 (4H, d, *J* = 7.2 Hz), 7.90 (4H, br), 7.32 (4H, br), 3.96 (6H, s). δ_C_ (CD_3_OD, 400 MHz, ppm): 162.48, 149.69, 145.35, 135.47, 126.87, 125.49, 115.41, 55.17.

Data for EV6Cl: Recrystallized from methanol/ethyl acetate, yield 81%. δ_H_ (CD_3_OD, 400 MHz, ppm): 9.49 (4H, d, *J* = 7.2 Hz), 8.79 (4H, d, *J* = 7.2 Hz), 7.89 (4H, br), 7.30 (4H, br), 4.15 (4H, t, *J* = 6.4 Hz), 1.81–1.87 (4H, m), 1.38–1.55 (12H, m), 0.96 (6H, t, *J* = 6.8 Hz). δ_C_ (CD_3_OD, 400 MHz, ppm): 161.94, 149.64, 145.30, 135.32, 126.88, 125.47, 115.85, 68.54, 31.30, 28.74, 25.36, 22.26, 12.98.

Data for EV8Cl: Recrystallized from methanol/ethyl acetate, yield 78%. δ_H_ (CD_3_OD, 400 MHz, ppm): 9.50 (4H, d, *J* = 7.2 Hz), 8.90 (4H, d, *J* = 7.2 Hz), 7.90 (4H, br), 7.31 (4H, br), 4.16 (4H, t, *J* = 6.4 Hz), 1.82–1.88 (4H, m), 1.33–1.53 (20H, m), 0.92 (6H, t, *J* = 6.8 Hz). δ_C_ (CD_3_OD, 400 MHz, ppm): 161.93, 149.64, 145.30, 135.32, 126.89, 125.48, 115.85, 68.54, 31.58, 29.04, 28.98, 28.78, 25.69, 22.31, 13.05.

Data for EV10Cl: Recrystallized from ethanol/ethyl acetate, yield 80%. δ_H_ (CD_3_OD, 400 MHz, ppm): 9.49 (4H, d, *J* = 7.2 Hz), 8.90 (4H, d, *J* = 7.2 Hz), 7.89 (4H, br), 7.30 (4H, br), 4.16 (4H, t, *J* = 6.4 Hz), 1.83–1.87 (4H, m), 1.32–1.53 (28H, m), 0.92 (6H, t, *J* = 6.8 Hz). δ_C_ (CD_3_OD, 400 MHz, ppm): 161.94, 149.64, 145.30, 135.31, 126.87, 125.46, 115.85, 68.54, 31.65, 29.31, 29.28, 29.07, 29.04, 28.78, 25.69, 22.32, 13.05.

Data for EV12Cl: Recrystallized from ethanol, yield 60%. δ_H_ (CD_3_OD, 400 MHz, ppm): 9.49 (4H, d, *J* = 7.2 Hz), 8.90 (4H, d, *J* = 7.2 Hz), 7.89 (4H, br), 7.30 (4H, br), 4.16 (4H, t, *J* = 6.4 Hz), 1.83–1.87 (4H, m), 1.32–1.53 (36H, m), 0.92 (6H, t, *J* = 6.8 Hz). δ_C_ (CD_3_OD, 400 MHz, ppm): 161.94, 149.64, 145.30, 135.31, 126.87, 125.46, 115.85, 68.54, 31.65, 29.31, 29.28, 29.07, 29.04, 28.78, 25.69, 22.32, 13.05.

Data for EV14Cl: Recrystallized from ethanol, yield 80%. δ_H_ (CD_3_OD, 400 MHz, ppm): 9.49 (4H, d, *J* = 5.6 Hz), 8.88 (4H, d, *J* = 6.0 Hz), 7.87 (4H, br), 7.30 (4H, br), 4.16 (4H, t, *J* = 6.4 Hz), 1.82–1.87 (4H, m), 1.30–1.54 (44H, m), 0.92 (6H, t, *J* = 6.8 Hz).

Data for EV16Cl: Recrystallized from ethanol, yield 82%. δ_H_ (CD_3_OD, 400 MHz, ppm): 9.50 (4H, d, *J* = 6.8 Hz), 8.88 (4H, d, *J* = 7.2 Hz), 7.87 (4H, br), 7.30 (4H, br), 4.14 (4H, t, *J* = 6.4 Hz), 1.87–1.78 (4H, m), 1.22–1.42 (52H, m), 0.91 (6H, t, *J* = 6.8 Hz).

Data for EV18Cl: Recrystallized from ethanol, yield 45%. δ_H_ (CD_3_OD, 400 MHz, ppm): 9.49 (4H, d, *J* = 7.2 Hz), 8.80 (4H, d, *J* = 7.2 Hz), 7.87 (4H, br), 7.30 (4H, br), 4.16 (4H, t, *J* = 6.4 Hz), 1.83–1.87 (4H, m), 1.29–1.54 (60H, m), 0.91 (6H, t, *J* = 6.8 Hz).

Data for EV20Cl: Recrystallized from ethanol, yield 45%. δ_H_ (CD_3_OD, 400 MHz, ppm): 9.50 (4H, d, *J* = 6.8 Hz), 8.87 (4H, d, *J* = 7.2 Hz), 7.87 (4H, br), 7.30 (4H, br), 4.16 (4H, t, *J* = 6.4 Hz), 1.82–1.87 (4H, m), 1.29–1.54 (68H, m), 0.91 (6H, t, *J* = 6.8 Hz).

### 3.6. Synthesis of bis(4-Methoxyphenyl)-4,4′-Bipyridinium Bis(triflimide) Salt (EV1)

A clear solution of 0.95 g (3.31 mmol) lithium triflimide in 5 mL water was added dropwise to the clear solution of 0.66 g (1.50 mmol) of EV1Cl in 25 mL water on stirring at room temperature. The precipitation occurred immediately in the reaction mixture and it was stirred overnight to complete the reaction. Then, the product was filtered, washed with plenty of water, and dried in a vacuum to yield 1.19 g (1.28 mmol) of the pure product EV1. Yield 85%. δ_H_ (CD_3_OD, 400 MHz, ppm): 9.44 (4H, d, *J* = 6.8 Hz), 8.80 (4H, d, *J* = 6.8 Hz), 7.84 (4H, br), 7.30 (4H, br), 3.95 (6H, s). δ_C_ (CD_3_OD, 400 MHz, ppm): 162.49, 149.87, 145.34, 135.26, 126.83, 125.44, 121.28, 118.09, 115.38, 55.13. Anal. calcd for C_28_H_22_N_4_O_8_F_12_S_4_ (930.74): C, 36.13; H, 2.38; N, 6.02; S, 13.78%. Found C, 36.42; H, 2.26; N, 6.09; S, 13.66%.

### 3.7. General Procedure for the Synthesis of Bis(4-n-alkoxyphenyl)-4,4′-bipyridinium Bis(triflimide) Salts (EV6, EV8, EV10, EV12, EV14, EV16, EV18, EV20) by Metathesis Reaction 

The following procedure [31,32,33], as an example, was adopted for the synthesis of EV20 from the metathesis reaction of EV20Cl with lithium triflimide. An amount of 0.33 g (0.34 mmol) EV20Cl was dissolved in methanol on boiling to get a clear solution to which clear methanol solution of lithium triflimide 0.22 g (0.75 mmol) dissolved in 5 mL was added on stirring. The reaction mixture was heated to reflux on stirring overnight for the completion of the metathesis reaction. The product precipitated out on cooling the reaction mixture to the ambient temperature. It was collected by filtration, washed with plenty of water, and recrystallized from methanol yielding 0.24 g (0.16 mmol). In the cases of EV6, EV8, EV10, and EV12, after the completion of the metathesis reaction, methanol was removed by rotatory evaporator to give the crude product that was repeatedly washed with water to give the pure product.

Data for EV6: Yield 67%. δ_H_ (CD_3_OD, 400 MHz, ppm): 9.43 (4H, d, *J* = 6.8 Hz), 8.79 (4H, d, *J* = 6.8 Hz), 7.83 (4H, br), 7.29 (4H, br), 4.15 (4H, t, *J* = 6.4 Hz), 1.81–1.87 (4H, m), 1.36–1.55 (12H, m), 0.96 (6H, t, *J* = 7.2 Hz). δ_C_ (CD_3_OD, 400 MHz, ppm): 161.97, 149.82, 145.29, 135.26, 126.82, 125.41, 121.28, 118.09, 115.82, 68.53, 31.28, 28.71, 25.33, 22.23, 12.93. Anal. calcd for C_38_H_42_N_4_O_10_F_12_S_4_ (1071.00): C, 42.61; H, 3.95; N, 5.23; S, 11.98%. Found C, 42.38; H, 3.38; N, 5.20; S, 12.27%.

Data for EV8: Yield 79%. δ_H_ (CD_3_OD, 400 MHz, ppm): 9.43 (4H, d, *J* = 7.2 Hz), 8.79 (4H, d, *J* = 7.2 Hz), 7.84 (4H, br), 7.29 (4H, br), 4.15 (4H, t, *J* = 6.4 Hz), 1.81–1.87 (4H, m), 1.33–1.54 (20H, m), 0.93 (6H, t, *J* = 7.2 Hz). δ_C_ (CD_3_OD, 400 MHz, ppm): 161.96, 149.82, 145.29, 135.26, 126.83, 125.41, 121.28, 118.09, 115.83, 68.52, 31.55, 29.01, 28.96, 28.75, 25.66, 22.28, 13.00. Anal. calcd for C_42_H_50_N_4_O_10_F_12_S_4_ (1127.11): C, 44.76; H, 4.47; N, 4.97; S, 11.38%. Found C, 44.54; H, 4.48; N, 4.92; S, 11.40%.

Data for EV10: Yield 80%. δ_H_ (CD_3_OD, 400 MHz, ppm): 9.43 (4H, d, *J* = 6.8 Hz), 8.79 (4H, d, *J* = 6.8 Hz), 7.82 (4H, br), 7.28 (4H, br), 4.15 (4H, t, *J* = 6.4 Hz), 1.81–1.88 (4H, m), 1.32–1.54 (28H, m), 0.92 (6H, t, *J* = 7.2 Hz). δ_C_ (CD_3_OD, 400 MHz, ppm): 161.96, 149.82, 145.28, 135.25, 126.83, 125.41, 121.28, 118.09, 115.83, 68.52, 31.64, 29.35, 29.26, 29.05, 29.02, 28.75, 25.66, 22.30, 13.02. Anal. calcd for C_46_H_58_N_4_O_10_F_12_S_4_ (1183.21): C, 46.69; H, 4.94; N, 4.71; S, 10.84%. Found C, 46.44; H, 4.84; N, 4.73; S, 10.73%.

Data for EV12: Yield 79%. δ_H_ (CD_3_OD, 400 MHz, ppm): 9.44 (4H, br), 8.79 (4H, br), 7.83 (4H, d, *J* = 8.8 Hz), 7.28 (4H, d, *J* = 8.8 Hz), 4.15 (4H, t, *J* = 6.4 Hz), 1.83–1.87 (4H, m), 1.31–1.54 (36H, m), 0.92 (6H, t, *J* = 7.2 Hz). δ_C_ (CD_3_OD, 400 MHz, ppm): 161.98, 149.82, 145.29, 135.25, 126.81, 125.40, 121.29, 118.09, 115.83, 115.75, 68.53, 31.64, 29.35, 29.32, 29.29, 29.04, 28.76, 25.66, 22.30, 13.01. Anal. calcd for C_50_H_66_N_4_O_10_F_12_S_4_ (1239.32): C, 48.46; H, 5.37; N, 4.52; S, 10.35%. Found C, 48.48; H, 5.32; N, 4.51; S, 10.20%.

Data for EV14: Recrystallized from ethanol, yield 72%. δ_H_ (*d_6_-*DMSO, 400 MHz, ppm): 9.60 (4H, d, *J* = 6.8 Hz), 8.98 (4H, d, *J* = 6.8 Hz), 7.88 (4H, br), 7.30 (4H, br), 4.11 (4H, t, *J* = 6.8 Hz), 1.73–1.76 (4H, m), 1.22–1.44 (44H, m), 0.85 (6H, t, *J* = 6.8 Hz). δ_C_ (*d_6_-*DMSO, 400 MHz, ppm): 161.39, 148.75, 146.04, 145.99, 135.52, 126.90, 126.59, 124.70, 121.50, 118.30, 116.11, 68.79, 31.73, 29.49, 29.48, 29.45, 29.20, 29.15, 28.93, 25.87, 22.53, 14.36. Anal. calcd for C_54_H_74_N_4_O_10_F_12_S_4_ (1295.43): C, 50.07; H, 5.76; N, 4.32; S, 9.90%. Found C, 50.61; H, 5.77; N, 4.31; S, 9.79%.

Data for EV16: Recrystallized from ethanol, yield 74%. δ_H_ (*d_6_-*DMSO, 400 MHz, ppm): 9.60 (4H, br), 8.98 (4H, br), 7.87 (4H, br), 7.29 (4H, br), 4.11 (4H, t, *J* = 5.6 Hz), 1.71–1.78 (4H, m), 1.22–1.42 (52H, m), 0.84 (6H, t, *J* = 6.4 Hz). δ_C_ (*d_6_-*DMSO, 400 MHz, ppm): 161.39, 148.75, 146.08, 146.03, 145.99, 135.52, 126.90, 126.59, 124.70, 121.50, 118.30, 116.11, 68.80, 31.73, 29.49, 29.48, 29.45, 29.20, 29.15, 28.93, 25.88, 22.52, 14.37. Anal. calcd for C_58_H_82_N_4_O_10_F_12_S_4_ (1351.53): C, 51.54; H, 6.12; N, 4.15; S, 9.49%. Found C, 51.63; H, 6.11; N, 4.06; S, 9.31%.

Data for EV18: Recrystallized from ethanol, yield 61%. δ_H_ (*d_6_-*DMSO, 400 MHz, ppm): 9.59 (4H, br), 8.98 (4H, br), 7.87 (4H, br) 7.29 (4H, br) 4.11 (4H, t, *J* = 6.4 Hz), 1.71–1.78 (4H, m), 1.22–1.42 (60H, m), 0.84 (6H, *J* = 6.4 Hz). δ_C_ (*d_6_-*DMSO, 400 MHz, ppm): 161.39, 148.76, 146.04, 145.99, 135.52, 126.90, 126.59, 124.70, 121.50, 118.30, 116.11, 68.79, 31.74, 29.48, 29.45, 29.40, 29.39, 29.38, 29.23, 29.15, 28.95, 25.89, 22.53, 14.34. Anal. calcd for C_62_H_90_N_4_O_10_F_12_S_4_ (1407.64): C, 52.90; H, 6.44; N, 3.98; S, 9.11%. Found C, 53.10; H, 6.53; N, 4.01; S, 8.99%.

Data for EV20: Recrystallized from methanol, yield 47%. δ_H_ (*d_6_-*DMSO, 400 MHz, ppm): 9.63 (4H, d, *J* = 6.4 Hz), 9.02 (4H, d, *J* = 6.4 Hz), 7.90 (4H, br) 7.33 (4H, br) 4. 14 (4H, t, *J* = 6.4 Hz), 1.75–1.79 (4H, m), 1.24–1.46 (68H, m), 0.87 (6H, *J* = 6.8 Hz). δ_C_ (*d_6_-*DMSO, 400 MHz, ppm): 161.38, 148.69, 146.01, 135.52, 126.87, 126.61, 124.70, 121.50, 118.30, 116.12, 68.80, 31.72, 29.47, 29.46, 29.44, 29.21, 29.13, 28.94, 25.88, 22.52, 14.38. Anal. calcd for C_66_H_98_N_4_O_10_F_12_S_4_ (1463.75): C, 54.16; H, 6.75; N, 3.83; S, 8.76%. Found C, 54.27; H, 6.60; N, 3.86; S, 8.87%.

## 4. Conclusions

A series of bis(4-alkoxyphenyl) viologen bis(triflimide) salts with alkoxy chains of different lengths were synthesized by the metathesis reaction of respective bis(4-alkoxyphenyl) viologen dichloride salts, which in turn prepared the reaction of Zincke salt with the corresponding 4-n-alkoxyanilines, with lithium triflimide in methanol. Their chemical structures were established using spectroscopic techniques and elemental analysis. Their thermotropic LC properties as determined by DSC, POM, and VT-XRD suggested that the salts with short alkoxy chains (*n* = 1 and 6 carbon atoms) did not form LC phase on melting, but the salts with long alkoxy chains (*n* = 8 and 10 carbon atoms) exhibited crystal–LC transitions, T_m_s, and LC–isotropic transitions, T_i_s, and showed Schlieren or focal conic textures indicative of their SmA phases. Those of higher alkoxy chains (*n* = 12, 14, 16, 18, and 20) exhibited SmA phases at relatively low T_m_s and their SmA phase persisted up to their decomposition at high temperatures. The majority of them also exhibited crystal-to-crystal transitions prior to the T_m_s. They had good thermal stability in the temperature range of 330–370 °C. These results suggest that they exhibited LC phases at relatively low T_m_s which have practical implications. Importantly, triflimide counterion is not only interesting for the preparation of ILs but also interesting for the preparation of ILCs, an important class of LC.

## Figures and Tables

**Table 1:** Thermodynamic properties of phase transition temperatures of extended viologen bis(triflimide) salts (EVn) obtained from DSC measurements and decomposition temperatures obtained from thermogravimetric analysis (TGA) measurements.

Sample	T_m_^a^ °C	T_m_^b^ °C	T_LC-LC_^c^ °C	T_i_^d^ °C	ΔT ^e^ °C	T_d_^f^ °C
EV1	104 ^g^ (17.4), 164 (25.8)	-	-	-	-	370
EV6	75 ^g^ (0.84), 105 (38.2)	-	-	-	-	328
EV8	-	104 ^g^ (4.6), 112 ^g^ (0.36), 122 (43.5)	-	190 (1.8)	68	330
EV10	-	104 (46.1)	123 (0.6)	251 (2.3)	147	332
EV12	-	104 (56.8)	130 (0.3)	305 ^h^ (3.4)	-	331
EV14	-	12 ^g^ (2.1), 40 ^g^ (5.5), 104 (55.9)	-	328 ^h^	-	337
EV16	-	54 ^g^ (1.0), 82 ^g^ (3.3), 101 (49.2)	-	-	-	337
EV18	-	63 ^g^ (2.4), 70 ^g^ (6.2), 106 (69.6)	-	-	-	331
EV20	-	71 ^g^ (2.9), 83 ^g^ (4.5), 107 (71.8)	-	-	-	330

^a^ T_m_ = crystal-to-isotropic phase transition. Datum was taken from the second heating cycle of the DSC thermogram at a heating rate of 10 °C/min. The value in the parentheses was the enthalpy in kJ/mol for this transition. ^b^ T_m_ = crystal-to-LC phase transition. Datum was taken from the second heating cycle of the DSC thermogram at a heating rate of 10 °C/min. The value in the parentheses was the enthalpy in kJ/mol for this transition. ^c^ T_LC-LC_ = LC-to-LC phase transition. ^d^ T_i_ = LC-to isotropic transition. Datum was taken from the second heating cycle of the DSC thermogram at a heating rate of 10 °C/min. The value in the parentheses was the enthalpy in kJ/mol for this transition. ^e^ ΔT = (T_i_-T_m_), that is, the LC phase range. ^f^ T_d_ = the temperature at which a 5% weight loss of the salt occurred at a heating rate of 10 °C/min in nitrogen. ^g^ Crystal-to-crystal transition. Datum was taken from the second heating cycle of the DSC thermogram at a heating rate of 10 °C/min. The value in the parentheses was the enthalpy in kJ/mol for this transition. ^h^ from reference [36].

## Supplementary Materials

The following are available online at https://www.mdpi.com/1420-3049/25/10/2435/s1, Figures S1–S5: ^1^H and ^13^C spectra of EV1Cl, EV6Cl, EV8Cl, EV10Cl, and EV12Cl, Figures S6–S9: ^1^H spectra of EV14Cl, EV16Cl, EV18Cl and EV20Cl, Figures S10–S18: ^1^H and ^13^C NMR spectra of EV1, EV6, EV8, EV10, EV12, EV14, EV16, EV18, and EV20, Figures S19–S23: DSC thermograms of EV6, EV12, EV16, EV18, and EV20, Figure S24: TGA thermograms of EV14, EV16, EV18, and EV20, Figures S25–S27: Emission spectra of EV1Cl and EV1, EV8Cl and EV8, and EV10Cl and EV10.
