# Supplementary material for: Thermotropic Liquid-Crystalline and Light-Emitting Properties of Bis(4-aalkoxyphenyl) Viologen Bis(triflimide) Salts
Source: Molecules. 2020 May 23;25(10):2435. doi: 10.3390/molecules25102435 (PMC7288076; doi:10.3390/molecules25102435)
Supplement: Supplementary file 1 [file molecules-25-02435-s001.pdf]

molecules

# Thermotropic Liquid-Crystalline and Light-Emitting Properties of bis(4-Aalkoxyphenyl) Viologen bis(Triflimide) Salts

**Pradip K. Bhowmik [1,*], Muhammed Kareem M. Al-Karawi [1], Shane T. Killarney [1], Erenz J. Dizon [1], Anthony Chang [1], Jongin Kim [1], Si L. Chen [1], Ronald Carlo G. Principe [1], Andy Ho [1], Haesook Han [1], Hari D. Mandal [2], Raymond G. Cortez [2], Bryan Gutierrez [2], Klarissa Mendez [2], Lewis Sharpnack [3], Deña M. Agra-Kooijman [4], Michael R. Fisch [5] and Satyendra Kumar [6]**

[1] Department of Chemistry and Biochemistry, University of Nevada Las Vegas, 4505 S. Maryland Parkway Box 454003, Las Vegas, NV 89154-4003, USA
[2] Department of Biology and Chemistry, Texas A & M International University, 5201 University Blvd., Laredo, TX 78041, USA
[3] Department of Earth Science, 1006 Webb Hall, University of California, Santa Barbara, CA 93106, USA
[4] Advanced Materials and Liquid Crystal Institute, Kent State University, Kent, OH 44242, USA
[5] College of Aeronautics and Engineering, Kent State University, Kent, OH 44242, USA
[6] Division of Research, University at Albany, Albany, NY 12222, USA
* Correspondence: pradip.bhowmik@unlv.edu; Tel.: +1 (702) 895-0885; +1 (702) 895-4072

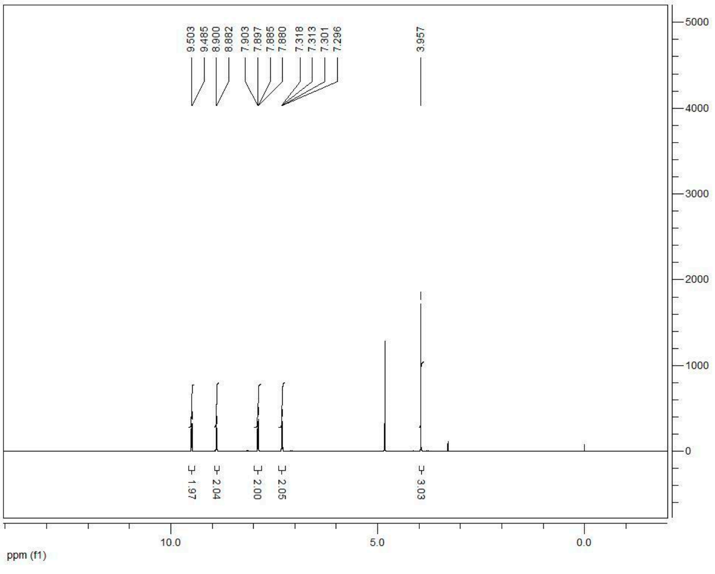


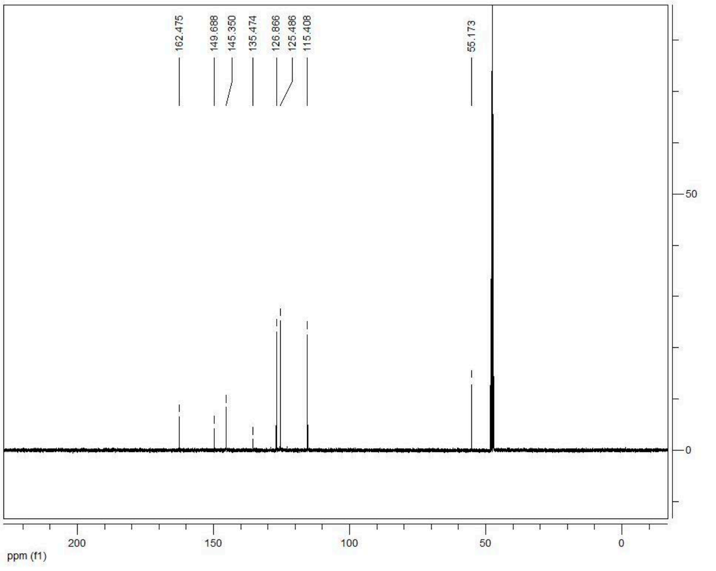


**Figure S1.** $^{1}H$ and $^{13}C$ NMR spectra of EV1Cl in $CD_3OD$ recorded at room temperature.

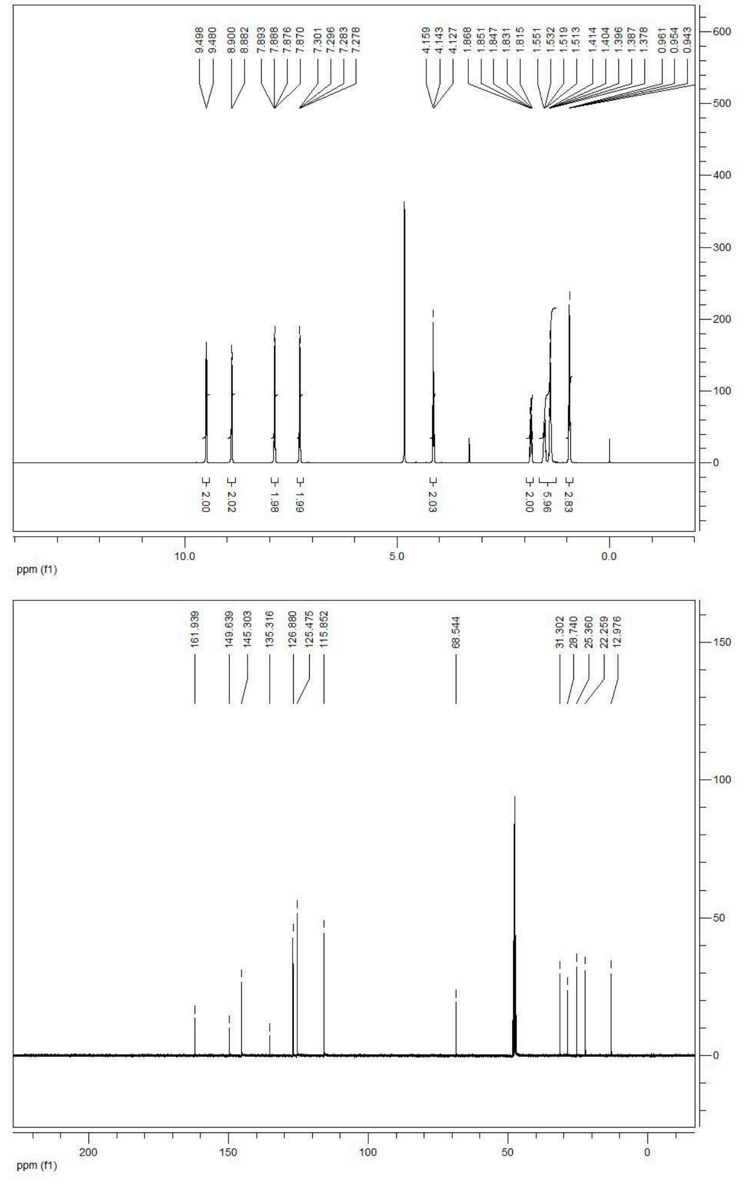

**Figure S2.** $^{1}H$ and $^{13}C$ NMR spectra of EV6Cl in $CD_3OD$ recorded at room temperature.

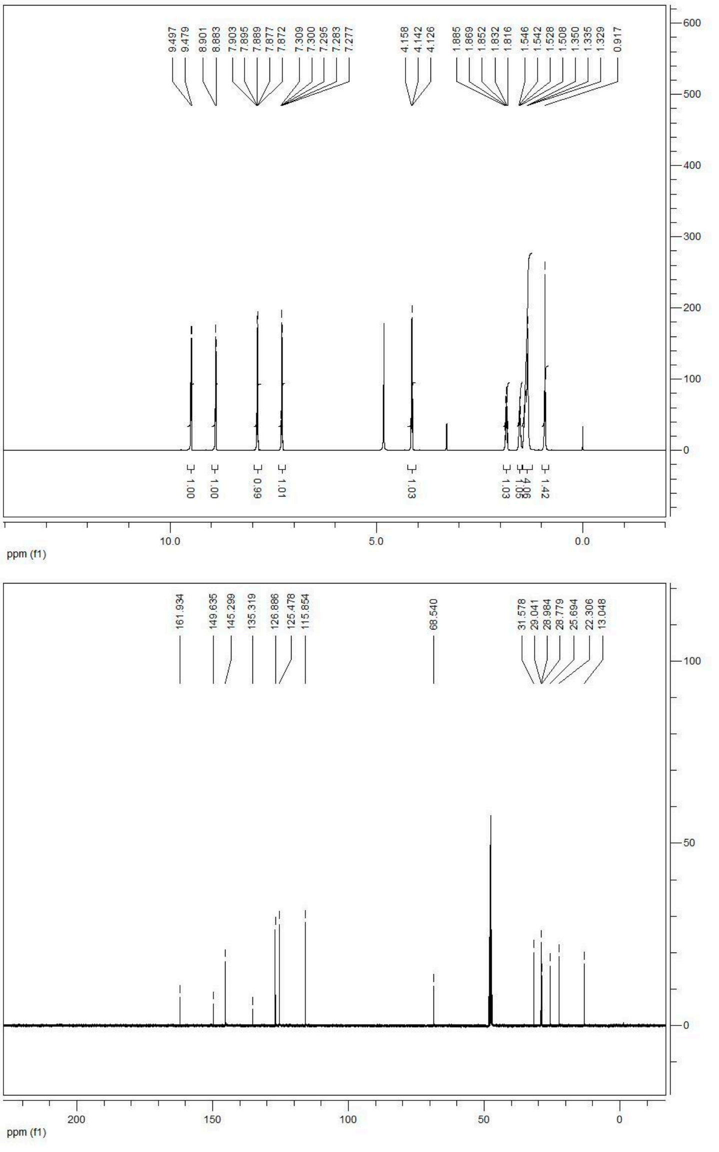

**Figure S3.** $^{1}H$ and $^{13}C$ NMR spectra of EV8Cl in $CD_3OD$ recorded at room temperature.

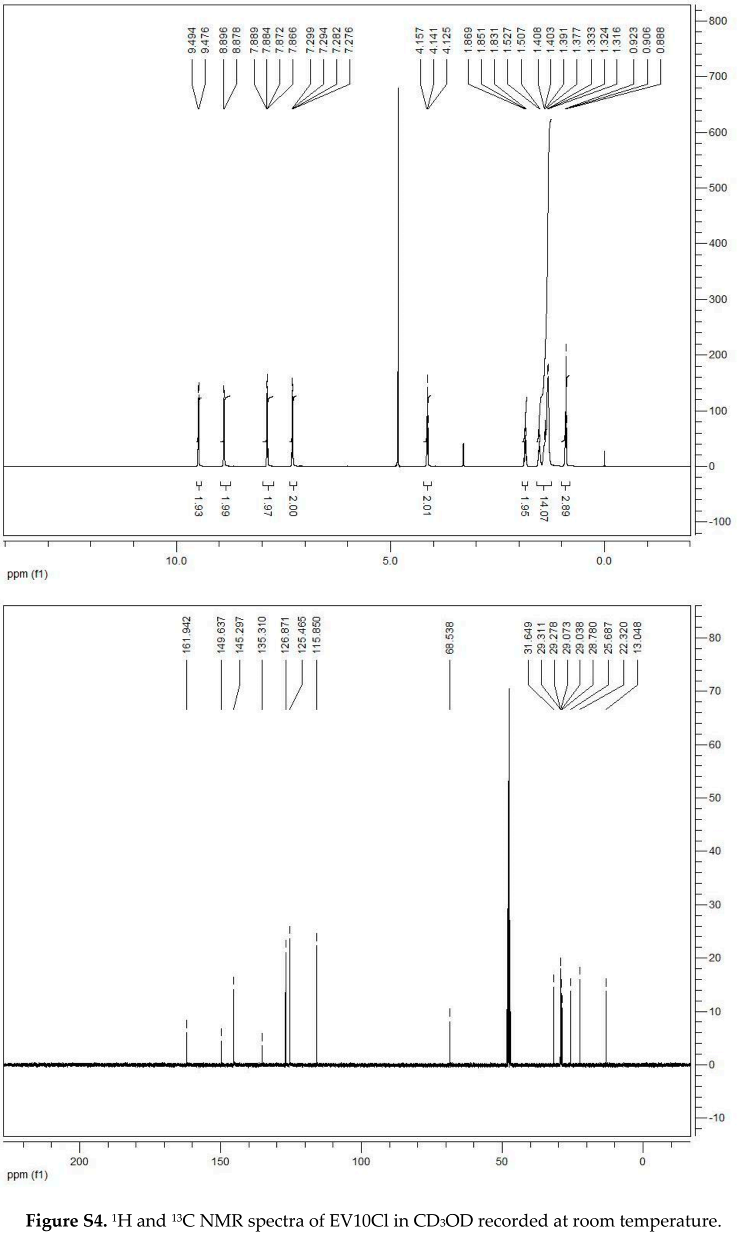


**Figure S4.** $^{1}H$ and $^{13}C$ NMR spectra of EV10Cl in $CD_3OD$ recorded at room temperature.

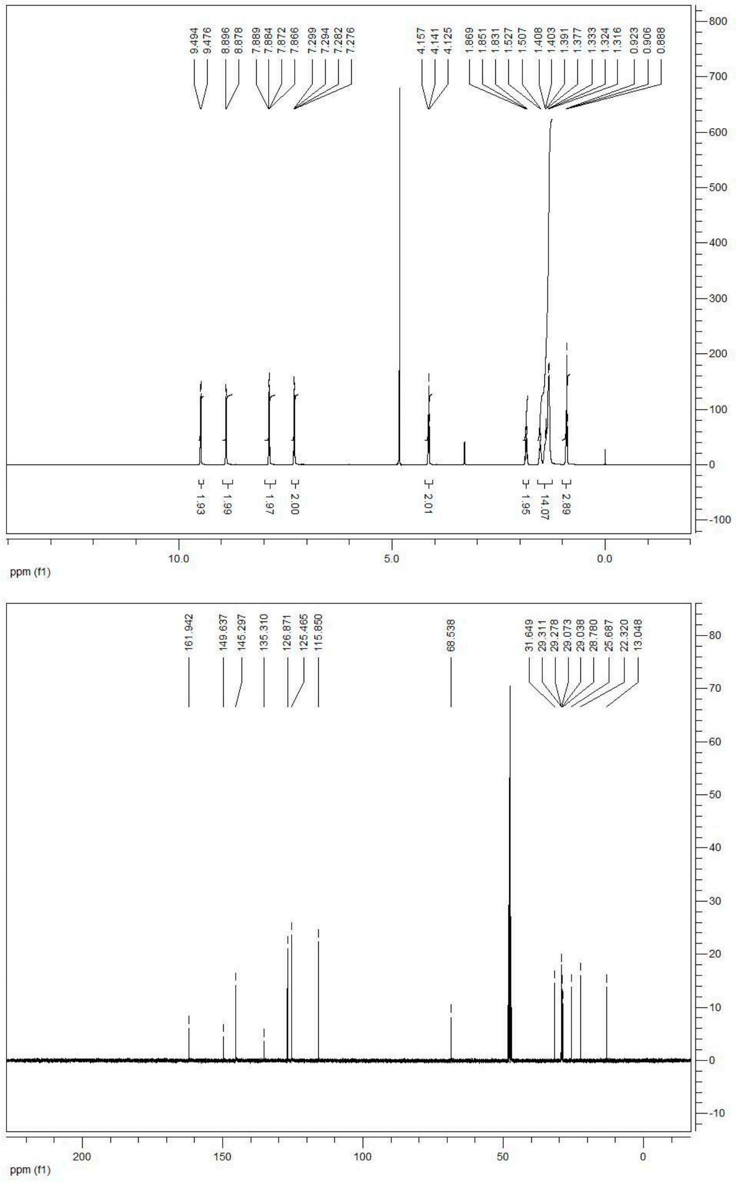


**Figure S5.** $^{1}H$ and $^{13}C$ NMR spectra of EV12Cl in $CD_3OD$ recorded at room temperature.

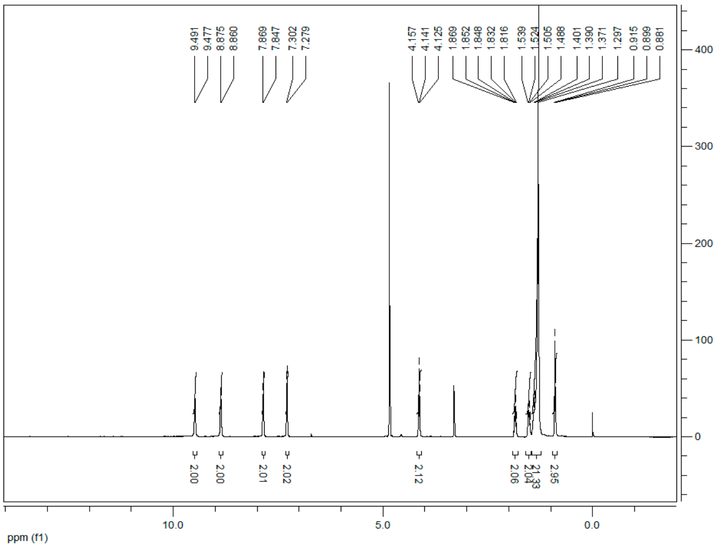

**Figure S6.** $^{1}H$ NMR spectrum of EV14Cl in $CD_3OD$ recorded at room temperature.

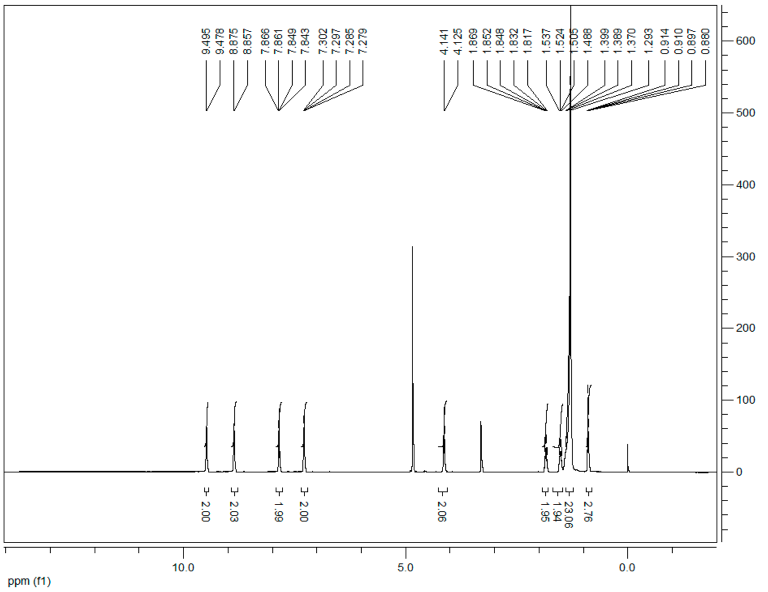

**Figure S7.** $^{1}H$ NMR spectrum of EV16Cl in $CD_3OD$ recorded at room temperature.

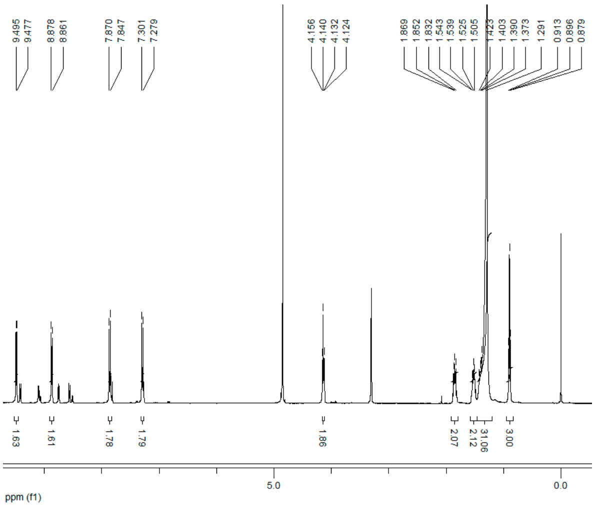

**Figure S8.** $^{1}$H NMR spectrum of EV18Cl in $CD_3OD$ recorded at room temperature.

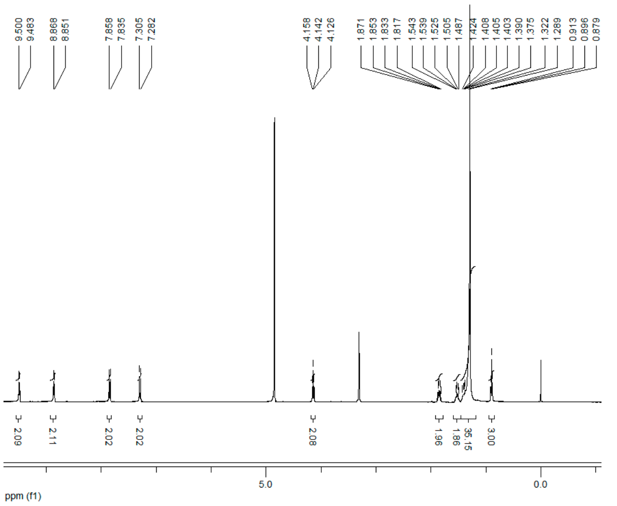

**Figure S9.** $^{1}$H NMR spectrum of EV20Cl in $CD_3OD$ recorded at room temperature.

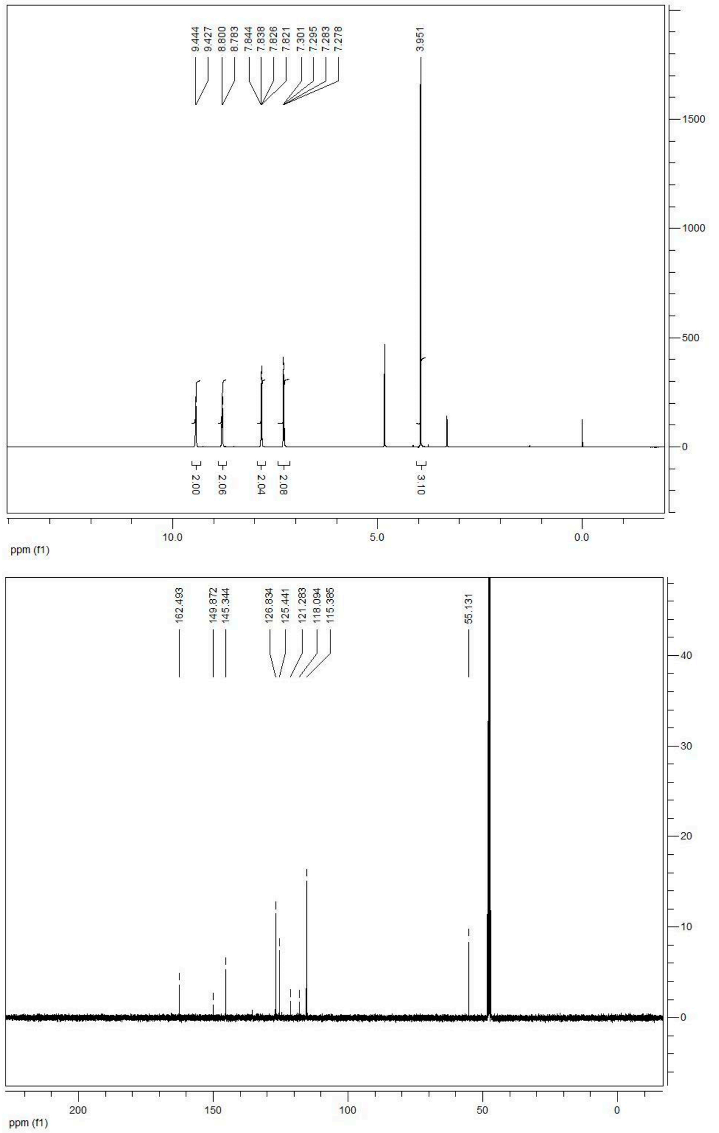


**Figure S10.** $^{1}H$ and $^{13}C$ NMR spectra of EV1 in $CD_3OD$ recorded at room temperature.

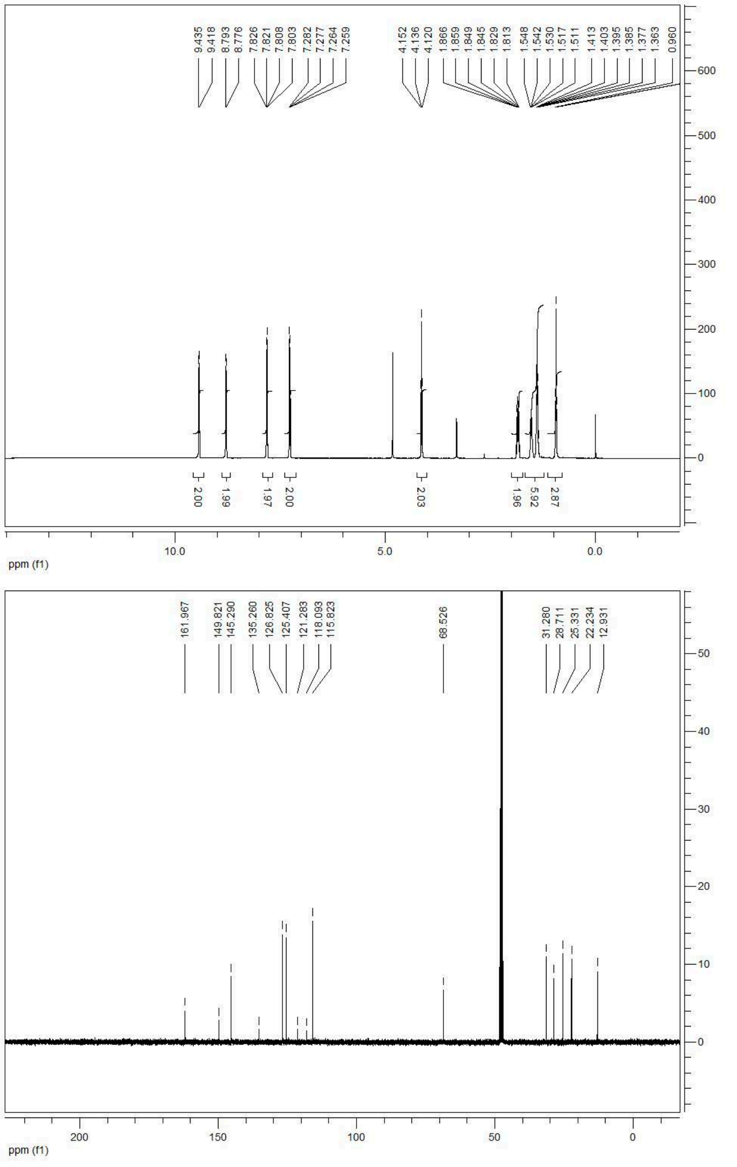


**Figure S11.** $^{1}H$ and $^{13}C$ NMR spectra of EV6 in $CD_3OD$ recorded at room temperature.

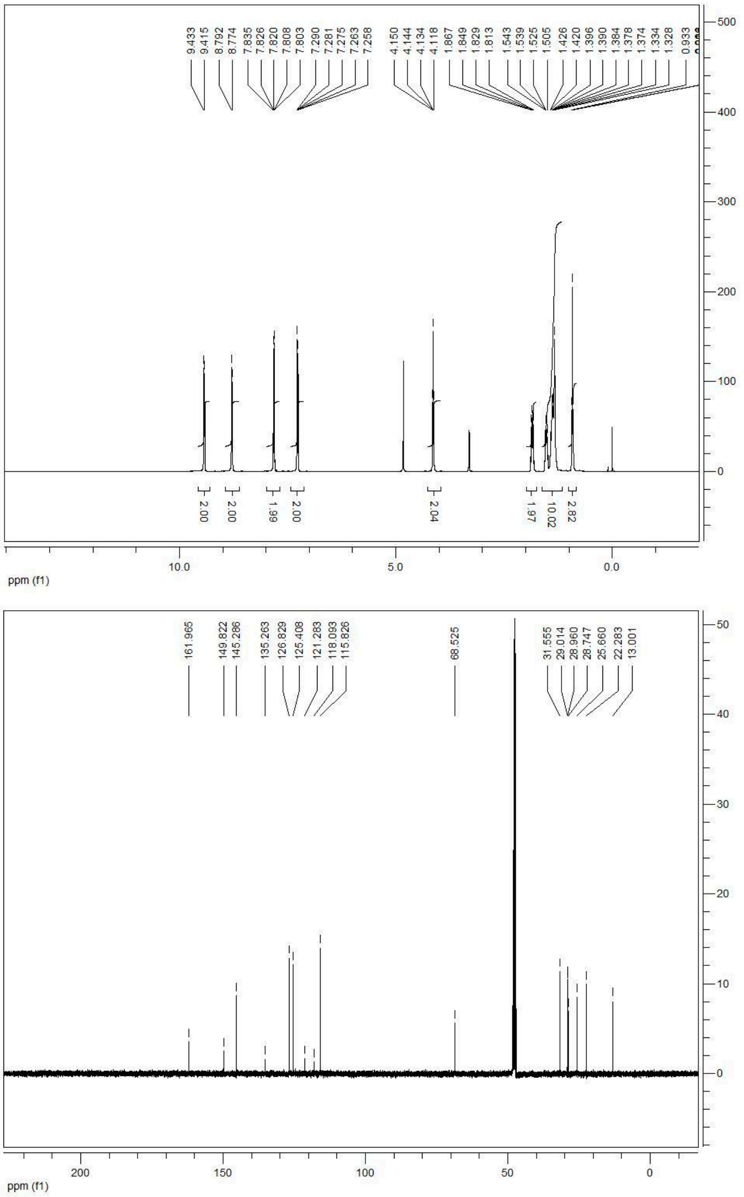


**Figure S12.** $^{1}H$ and $^{13}C$ NMR spectra of EV8 in $CD_3OD$ recorded at room temperature.

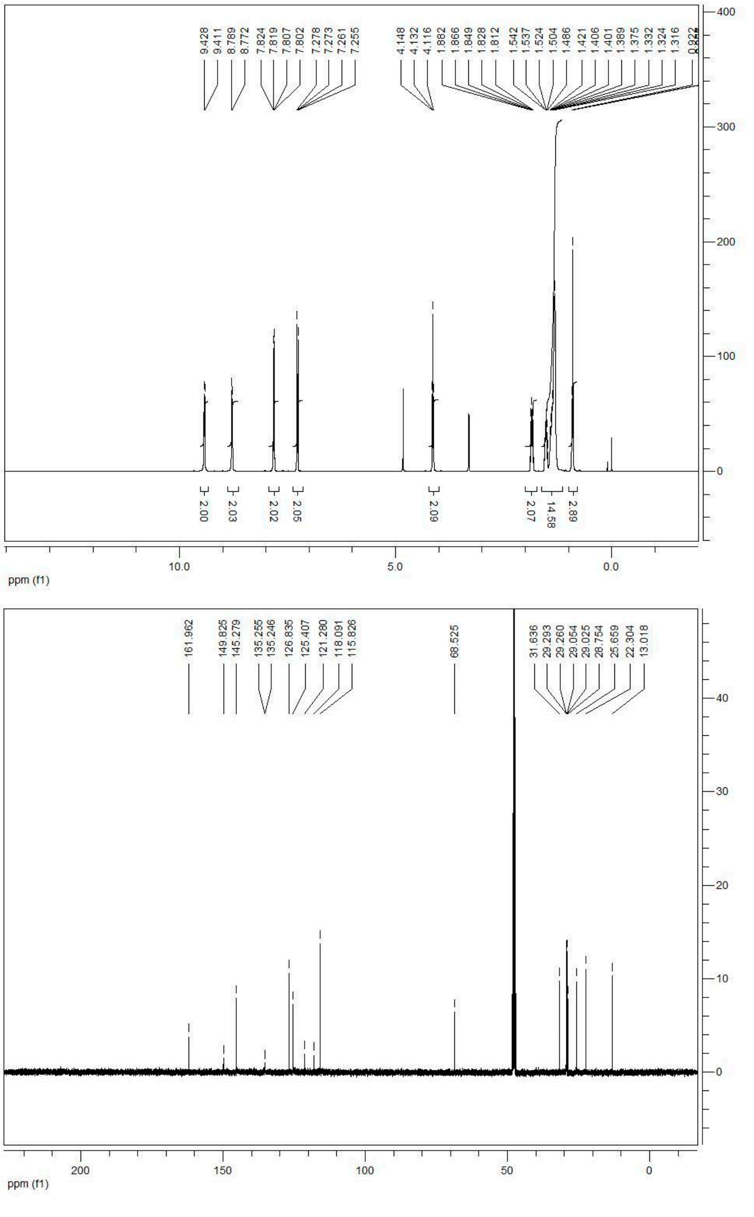


**Figure S13.** $^{1}H$ and $^{13}C$ NMR spectra of EV10 in $CD_3OD$ recorded at room temperature.

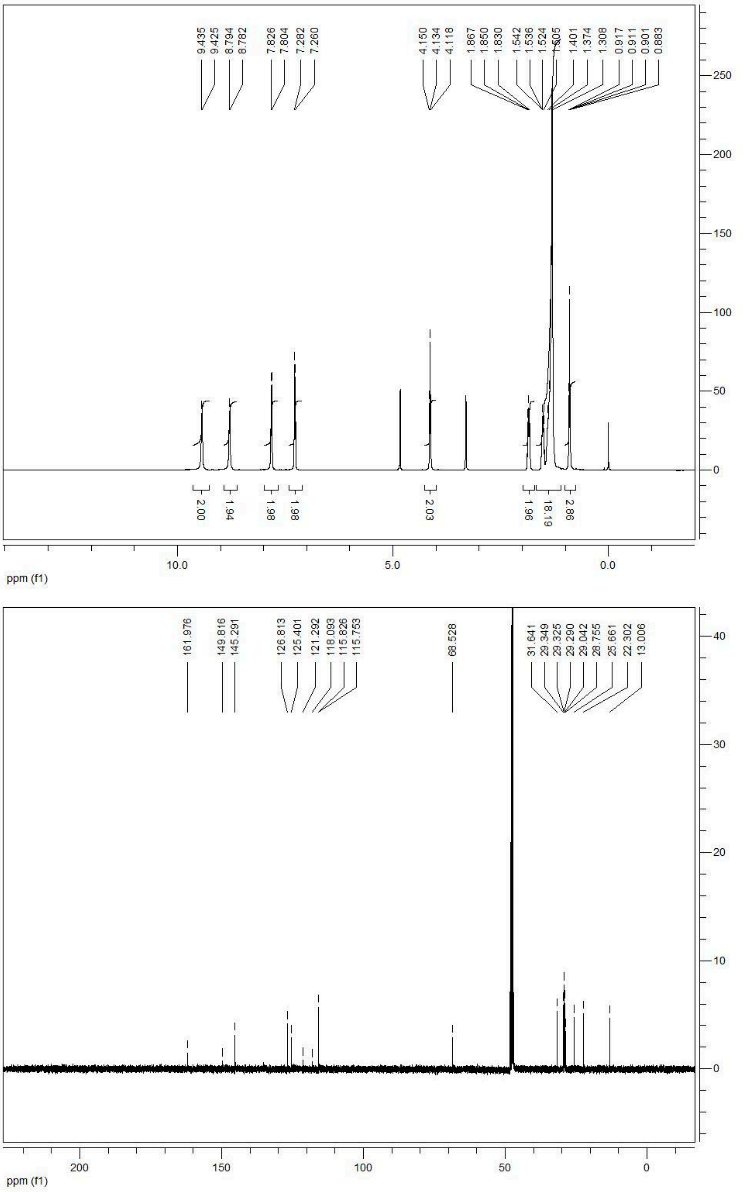

**Figure S14.** $^{1}H$ and $^{13}C$ NMR spectra of EV12 in $CD_3OD$ recorded at room temperature.

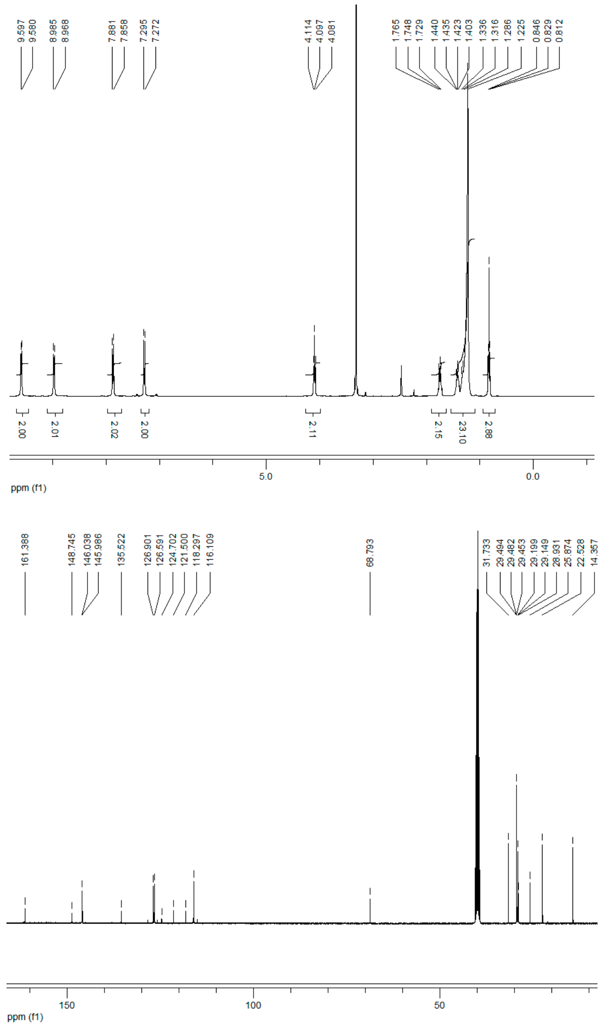


**Figure S15.** $^{1}H$ and $^{13}C$ NMR spectra of EV14 in *d6*-DMSO recorded at room temperature.

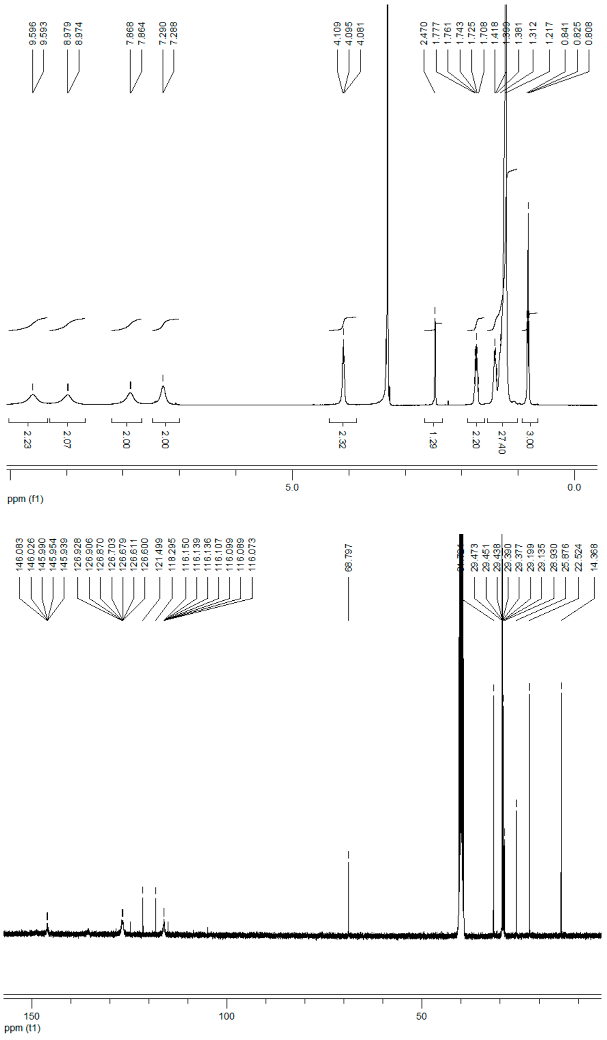


**Figure S16.** $^{1}H$ and $^{13}C$ NMR spectra of EV16 in *d6*-DMSO recorded at room temperature.

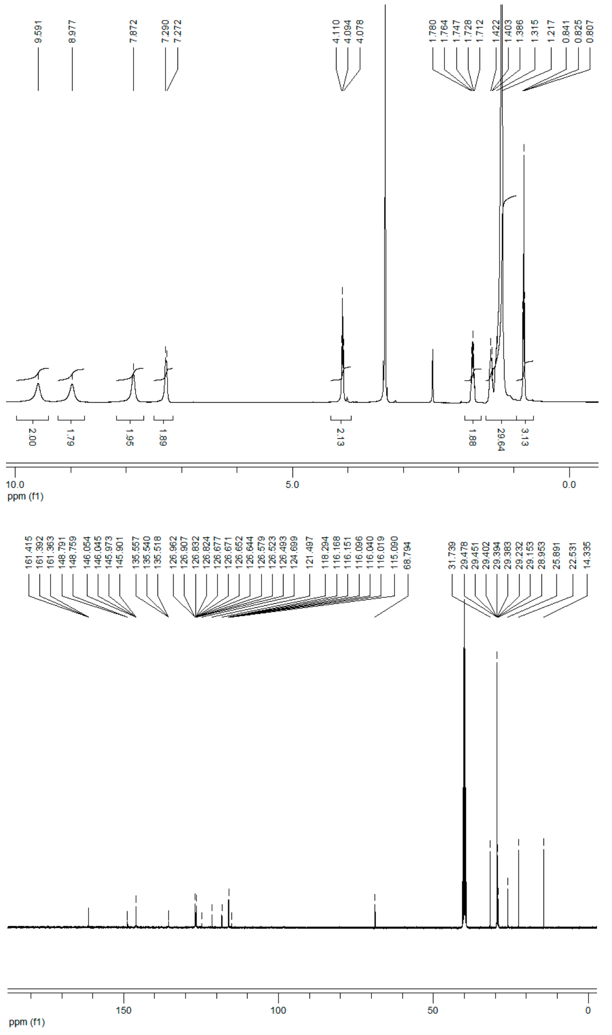

**Figure S17.** $^{1}H$ and $^{13}C$ NMR spectra of EV18 in $d_6$-DMSO recorded at room temperature.

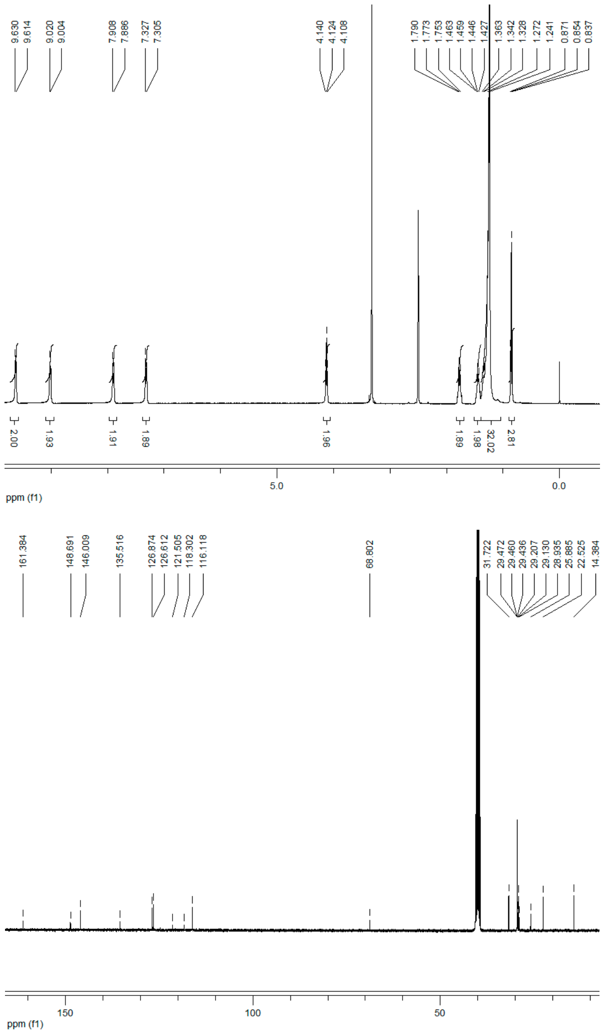


**Figure S18.** $^{1}H$ and $^{13}C$ NMR spectra of EV20 in $d_6$-DMSO recorded at room temperature.

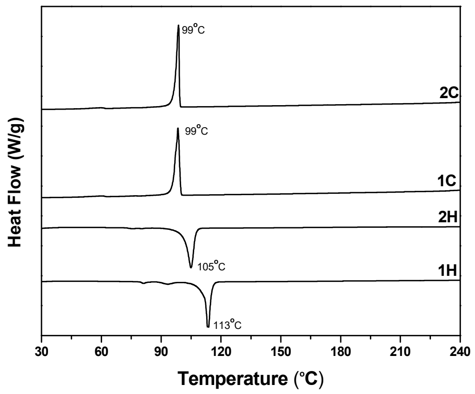


**Figure S19.** DSC thermograms of **EV6** obtained at heating and cooling rates of 10 °C /min.

**Commented [M1]:** Please add space between number and °.

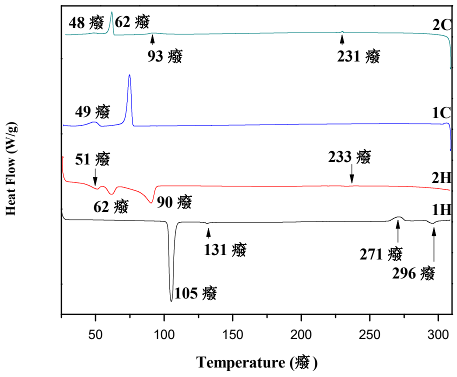


**Figure S20.** DSC thermograms of **EV12** obtained at heating and cooling rates of 10 °C /min.

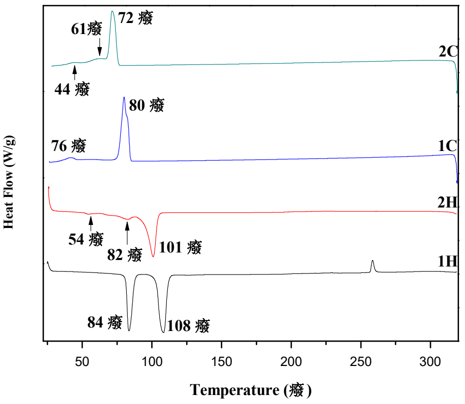


**Figure S21.** DSC thermograms of **EV16** obtained at heating and cooling rates of 10 °C /min.

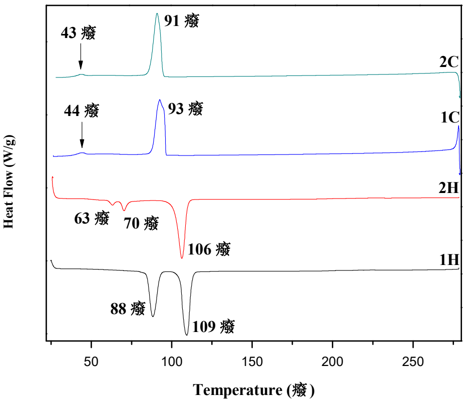


**Figure S22.** DSC thermograms of **EV18** obtained at heating and cooling rates of 10 °C /min.

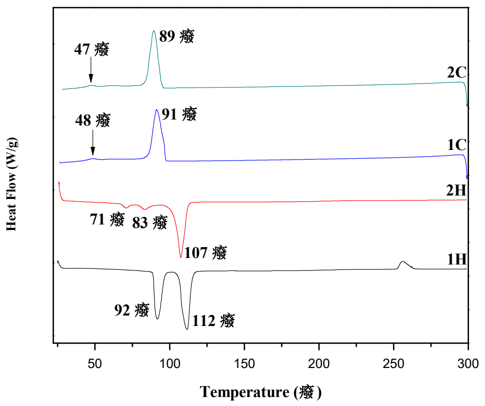


**Figure S23.** DSC thermograms of **EV20** obtained at heating and cooling rates of 10 °C /min.

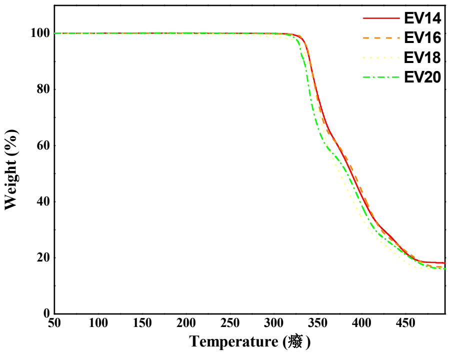


**Figure S24.** TGA thermograms of **EV14-EV20** obtained a heating rate of 10 °C/min in nitrogen.

(a)

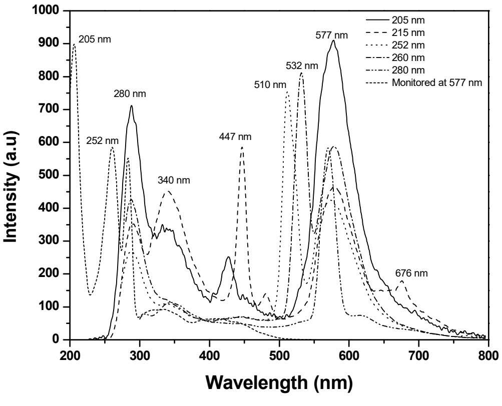


(b)

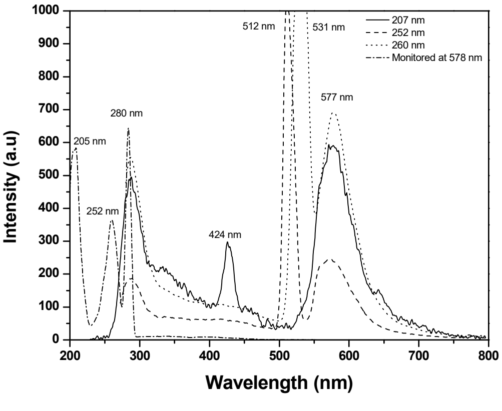


**Figure S25.** Emission spectra of (**a**) **EV1Cl** (**b**) **EV1** in methanol at various excitation wavelengths.

(a)

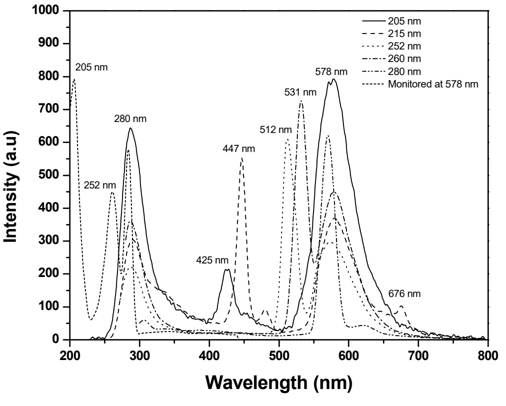


(b)

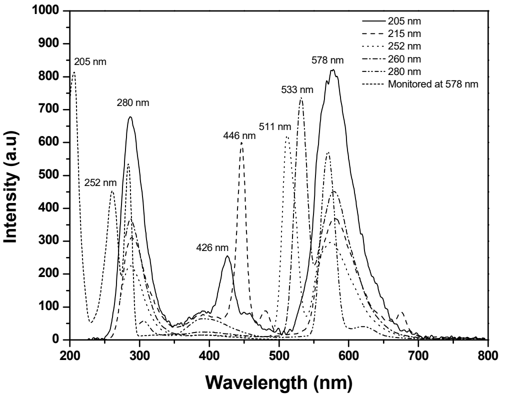


**Figure S26.** Emission spectra of (**a**) **EV8Cl** (**b**) **EV8** in methanol at various excitation wavelengths.

(a)

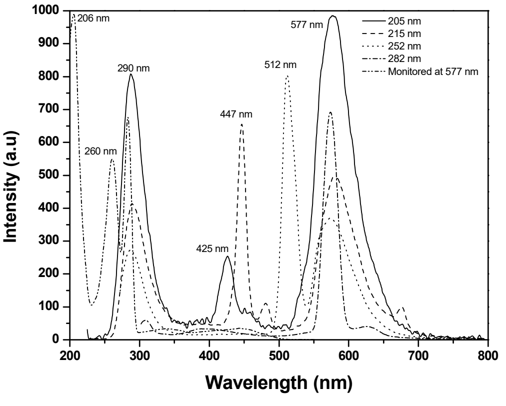


(b)

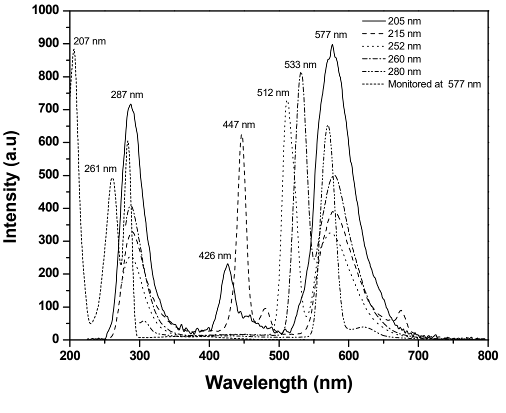


**Figure S27.** Emission spectra of (**a**) **EV10Cl** (**b**) **EV10** in methanol at various excitation wavelengths.
